# Study on the Effect of Microwaved Brewer’s Spent Grains on the Quality and Flavor Characteristics of Bread

**DOI:** 10.3390/foods13030461

**Published:** 2024-02-01

**Authors:** Jieyi Cheng, Li Zheng, Jinling Zhao, Meihong Yu, Rui Cao, Dan Wang, Jian Li, Linyi Zhou

**Affiliations:** 1 College of Food Science, Beijing Technology and Business University, Beijing 100048, China; cc20211028@163.com (J.C.); 15110631956@163.com (J.Z.); yumeihong8961@163.com (M.Y.); caorui13453422518@163.com (R.C.); wangdan1191@163.com (D.W.); lijian@th.btbu.edu.cn (J.L.); 2 College of Food Science, Northeast Agricultural University, Harbin 150030, China; 14794306051@163.com

**Keywords:** BSG, bread, microwave treatment, protein, dietary fiber, electronic nose, sensory evaluation

## Abstract

To enable a wider utilization of co–products from beer processing and minimize the negative effect of added grain on bread quality, flavor, and other attributes, brewer’s spent grains (BSG) are processed through microwave pretreatment, and then the microwave–treated BSG (MW–BSG) is added to bread. So far, there has been no investigation on the effect of microwave–pretreated BSG on bread quality and flavor. In this study, we examined the effects of diverse microwave treatment variables on the physicochemical structure of BSG and explored the consequences of MW–BSG on the quality and flavor of bread. The results showed that soluble dietary fiber and water–soluble protein levels in MW–BSG increased significantly (144.88% and 23.35%) at a 540 W microwave power, 3 min processing time, and 1:5 material–liquid ratio of BSG to water. The proper addition of MW–BSG positively affected the bread texture properties and color, but excessive amounts led to an irregular size and distribution of the bread crumbs. The result of electronic nose and HS–SPME–GC–MS analyses showed that the addition of MW–BSG modified the odor profile of the bread. A sensory evaluation showed mean scores ranging from 6.81 to 4.41 for bread containing 0–10% MW–BSG. Consumers found a maximum level of 6% MW–BSG acceptable. This study endeavors to decrease environmental contamination caused by brewing waste by broadening the methods by which beer co–products can be utilized through an innovative approach.

## 1. Introduction

Brewer’s spent grain (BSG) is the primary co–product of beer crafting, consisting primarily of the wet malt residue remaining after saccharification, with global production exceeding 180 million tons annually [1,2]. It has been estimated that approximately 30% of BSG is usually employed as animal feed with a relatively insignificant market value of around EUR 35 per ton. The remaining 70% of BSG is usually dumped into landfills by breweries. However, the disposal of this material in such a manner incurs costs of EUR 75–100 per ton and also results in the emission of considerable quantities of greenhouse gases, which have an adverse effect on the environment [3]. Furthermore, BSG contains a diverse range of nutrients and bioactive antioxidants, with protein and dietary fiber being the significant constituents. Cereal products have lysine as the limiting essential amino acid, which is present in approximately 30% of the total protein content of BSG [4]. Dietary fiber is considered to be a possible beneficial substance in regulating the composition of the gut microbiota and promoting the production of short–chain fatty acids [5]. Consequently, incorporating BSG into food products not only diminishes resource waste but also enhances brewery economics in a novel manner.

Bread is a commonly consumed staple food with a high demand from consumers. It is primarily made from processed wheat flour, which has a high glycemic index and may not be suitable for patients with obesity, diabetes, and hypertension [6]. Moreover, eliminating bran and germs in the wheat flour refining process leads to a considerable decline in the grain’s nutritional value [7]. Some studies have shown that regular white bread does not meet the growing health needs of consumers due to its low nutrient content, including vitamins, minerals, and polyphenols [8]. Therefore, incorporating BSG into bread production can reduce the use of refined wheat flour, enhance the nutritional value of bread, and lower production costs. This process also increases the utilization of BSG and reduces the generation and accumulation of co–products.

Often used as animal feed or directly discarded with minimal use of resources, BSG is increasingly being explored by scientists as a green additive to wheat products to enhance their sustainability [9]. However, the inherent composition of BSG can affect the quality of the final product, resulting in smaller and harder wheat products due to its high dietary fiber content [10]. To mitigate the negative effect of BSG on the color and flavor of wheat products, some scientists have attempted to improve the amount of the physicochemical constituents of BSG through pretreatment. For example, Ye et al. [5] pretreated BSG with impingement drying, which resulted in a significant reduction in moisture content and an increase in the protein and dietary fiber content of BSG. The results demonstrated that consumers received BSG muffins well, which were higher in protein and exhibited brighter colors.

Microwave treatment is a commonly used contemporary technology in heat treatment. As a result of microwave heat penetrating the cell wall of grains, their structure is altered, which subsequently affects the composition and concentration of dietary fiber and other active ingredients [11]. Furthermore, microwave treatment is energy efficient and can be widely used as an environmentally friendly heating method [12]. However, no information is available on the effect of microwave–treated BSG (MW–BSG) on bread quality.

The aim of this research was to investigate the effects of microwave treatment on the primary components of BSG and the subsequent effects of microwave–treated BSG on bread quality (including specific volume, texture, color, microstructure, flavor, and sensory evaluation). For the first time, the present work included BSG as an ingredient by directly mixing it into the bread dough. In comparison to other methods of incorporating BSG, such as hot–air drying and freeze–drying followed by grinding, the current method is more straightforward and environmentally friendly, and it preserves the original nutrient content of the BSG to the highest extent possible. All of these investigations will help reduce the environmental effect of brewing while enhancing the value of BSG in bread production.

## 2. Materials and Methods

### 2.1. Materials and Reagents

Brewer’s spent grain (BSG) was provided by Tsingtao Brewery Co., Ltd. (Beijing, China). Angel’s active dry yeast (Yichang, China), Westgold’s unsalted butter (Hokitika, New Zealand), high–gluten bread flour (protein 13.5%, fat 0.7%, carbohydrates 69.9%, dietary fiber 3%), salt, caster sugar, eggs, and millet were purchased from local supermarket. 2–methyl–3–heptanone (99%) was obtained from Sigma–Aldrich (St. Louis, MO, USA). Hexane (>99%), diethyl ether (>99%), ethanol (>99%), sulfuric acid (70%), hydrochloric acid (37%), sodium hydroxide (97%), and boric acid (>99%) were purchased from Aladdin Biochemical Technology Co., Ltd. (Shanghai, China).

### 2.2. Determination of the Composition of BSG

Moisture content was determined using the direct drying method outlined in AOAC 934.01 [13]. Ash content was figured out by burning in a crucible, as per the AOAC 942.05 method [14]. Solvent extraction was used to measure crude fat, following AOAC 945.16 [13]. The contents of insoluble, soluble, and total dietary fiber (IDF, SDF, and TDF) were measured using the AOAC 991.43 method [15]. The Bradford method was used to determine the water–soluble protein content. The crude protein level was determined with the AOAC 2001.11 method [13], using 5.83 as a nitrogen–to–protein conversion factor. The experiment was conducted three times.

### 2.3. Microwave Treatment

Microwaves use electromagnetic radiation to generate heat by causing intermolecular friction within food. This affects the structure and properties of the food, resulting in fast and convenient cooking. Microwave treatment factors, including time, power, and material–liquid ratio, were tested in single–factor experiments using a microwave oven (GalanZ G90F25CN3LN–C2(T1), Guangzhou, China) to determine their effects on the basic composition of BSG. The microwave power was adjusted by selecting low, medium, and high heating modes corresponding to 40%, 60%, and 80% of the rated power (900 W). The BSG was mixed with water at different material–liquid ratios (1:3, 1:5, and 1:7) for 5 min using a chopper (Bear QSJ–B02X5, Foshan, China). The experimental parameters of each factor were as follows: when the microwave time was set at 3 min and the material–liquid ratio was 1:5, the microwave powers were varied at 360 W, 540 W, and 720 W. Similarly, for a microwave power of 540 W and a material–liquid ratio of 1:5, the microwave times were varied at 1 min, 3 min, and 5 min. For a microwave time of 3 min and a microwave power of 540 W, the material–liquid ratios were varied at 1:3, 1:5, and 1:7. The determination of all MW–BSG and BSG base components was repeated three times.

### 2.4. Preparation of Bread

The microwave treatment parameters were set at 540 W, 3 min, and 1:5 material–liquid ratio based on the results of the single–factor experiments. The microwave–treated BSG (MW–BSG) was then added to the bread. The method was as follows: the BSG was mixed with water at a material–liquid ratio of 1:5 for 5 min using a chopper. The resulting BSG slurry was then processed in the microwave power mode at 540 W for 3 min. Finally, the MW–BSG was added at 0, 2, 4, 6, 8, and 10% of wheat flour (120 g). Besides wheat flour, the other ingredients were 1.4 g salt, 16 g caster sugar, 12 g whole egg, 10 g melted butter, 2 g yeast, and 60 g purified water. The dough was kneaded for 15 min and then fermented in a steam oven (Midea PS20C1, Foshan, China) at 30 °C for 30 min. The dough was then evenly divided into 60 g portions and fermented again at 35 °C for 30 min. Finally, the dough was placed in a preheated steam oven and baked at 170 °C for 15 min to prepare the bread. The bread (dinner rolls) was cooled to room temperature for testing.

### 2.5. Determination of Bread Specific Volume

The specific volume of the bread was determined according to the method of Chisenga et al. [16], which was modified. The bread volume was measured using the millet substitution method with an accuracy of 0.1 mL. The specific calculation formula used was

(1) $$Bread\;specific\;volume\left(mL/g\right)=Bread\;volume/\mathrm{Bread}\;\mathrm{weight}$$

with an average value taken after three repetitions of each measurement repeat.

### 2.6. Determination of the Texture Properties of Bread

Textural properties of bread were determined according to the method of Aljobair [17] and modified. The bread was cut into thin slices with a thickness of 1 cm, and samples of 20 mm × 20 mm in the center of the bread slice were measured with a texture analyzer (XT–Plus C, Stable Micro Systems Ltd., London, UK). The texture properties of the bread slices, including hardness, springiness, cohesiveness, gumminess, chewiness, and resilience, were quantified using a P/36R probe. The measurements were taken at a pre–measurement speed of 2 mm/s, a medium speed of 1 mm/s, and a post–measurement speed of 2 mm/s. A compression ratio of 60%, a pressure of 5 g, and a compression interval time of 5 s were applied.

### 2.7. Determination of the Color of Bread Crust and Crumb

The CM–5 spectrophotometer (Konica Minolta, Japan) was used to measure the color intensity of bread crust and bread crumb; the measurement aperture was 10 mm × 10 mm; the whiteboard correction value was L* = 82.01, a* = 0.99, b* = −7.18; the total color difference was expressed in △E; and the calculation formula was

(2) $$△E=\sqrt{{△L*}^{2}+{△a*}^{2}+{△b*}^{2}}=\;\sqrt{{(L_{0}-L_{1})*}^{2}+{(a_{0}-a_{1})*}^{2}+{(b_{0}-b_{1})*}^{2}}$$

Among them, △L* represents the black and white difference (0 = black, 100 = white); △a* represents the red–green difference (+reddish, −green); △b* represents the yellow–blue difference (+yellow, −blue); L_0_, a_0_, b_0_ are 0% added bread chromaticity values; L_1_, a_1_, b_1_ are the chromaticity values of BSG bread in each group; and all measurements were repeated three times to take the average [6].

### 2.8. Determination of the Microstructure of Bread

The microstructure of bread was characterized using a field emission scanning electron microscope (SU8020, HITACHI, Tokyo, Japan). All samples were lyophilized using a vacuum freeze dryer (LGJ–12, SIHUANDONGGAN, Beijing, China) before measurement, and the lyophilized samples were taken and processed into slices of size 8 mm × 8 mm × 5 mm, placed on a sample table, and fixed with a liquid conductive adhesive; then, the samples were gold–sprayed using an ion sputterer (MC1000, HITACHI, Tokyo, Japan). The analysis was performed under high–vacuum conditions at an accelerating voltage of 5 kV and magnifications of 50×, 500×, and 1500× [18].

### 2.9. Analysis of the Flavors of Bread

#### 2.9.1. Electronic Nose (E–Nose)

The bionic electronic nose (PEN3, AIRSENSE, Schwerin, Germany) was determined with the direct headspace inspiration method: 3 g of the sample was accurately weighed and placed in a 40 mL headspace bottle. The sealed bottle was then equilibrated in a 35 °C water bath for 15 min before inserting an E–nose probe to measure the aroma components of the bread. E–nose measurement parameters: sampling interval 1 s, rinsing time 100 s, zero adjustment time 8 s, resampling time 5 s, measurement time 260 s, carrier gas flow rate 300 mL/min, injection flow rate 300 mL/min. The response value of the sensor tended to be stable after 255 s; information was acquired at 256, 257, and 258 s; each group of samples was parallelized 5 times; and the results were selected 3 times for data analysis [19]. The types of response substances corresponding to the different sensors of the PEN3 E–nose are shown in Supplementary Materials Table S1 [20].

#### 2.9.2. Volatile Compound Determination

Headspace solid–phase microextraction coupled with gas chromatography–mass spectrometry (HS–SPME–GC–MS) was used to analyze the composition and relative levels of volatile aroma compounds in bread crust and crumb. A total of 2.0 g of crust or crumb was weighed into a 20 mL headspace vial and compacted, 2–methyl–3–heptanone (0.0816 μg/μL, 5 μL) was added as an internal standard, and the headspace vial was equilibrated at 60 °C for 15 min and then extracted using an SPME extraction head (50/30 μm DVB–CAR–PDMS, Supelco, Bellefonte, PA, USA) for 40 min. The extractions were performed using gas chromatography. The volatile compounds were separated via GC–MS (7890B/5977A, Agilent, Palo Alto, CA, USA) using a 60 m × 0.25 mm, 0.25 μm DB–WAX capillary column (Agilent, Palo Alto, CA, USA), with a ramp–up procedure of holding at 40 °C for 2 min, then ramping up to 90 °C at a rate of 4 °C/min, holding for 2 min, and then ramping up again to 140 °C at a rate of 2 °C/min. The mass spectrometry conditions were as follows: electron impact ionization (EI), electron energy 70 ev, inlet temperature 250 °C, interface temperature 250 °C, ion source temperature 230 °C, quaternary column temperature 150 °C, solvent delay 6 min, full scan mode, and mass scan range 50–500 amu. The peak area normalization method was used to calculate the relative percentage content of each compound in different samples [21]. The formula is shown in (3):

(3) $$C_{1}=\frac{\;A_{1}*C_{0}*V}{M*A_{0}}$$

where C_1_ and C_0_ denote sample concentration (ng/g) and internal standard concentration (μg/μL), respectively; A_1_ and A_0_ denote sample peak area and internal standard peak area, respectively; V denotes internal standard volume (μL), and M denotes sample mass (g).

### 2.10. Sensory Evaluation

The sensory evaluation team consisted of 10 experienced food students (age 20~30 years, male to female ratio 4:6). Each sample provided for the sensory evaluation was a single bread baked on the same day, complete single bread, and bread slices with a thickness of about 10 mm, which were evaluated for the crust color, crust texture, crumb color, crumb structure, taste, aroma, mouthfeel, and overall preference of the bread; the scoring rules and scuffing ranges are shown in Supplementary Materials—Table S2 [22], and the overall preference was expressed by the 9–point scale method. Members of the sensory evaluation team relieved fatigue by drinking purified water between the tastings of each set of samples. To avoid the subjective influence of the addition amount on the sensory experiment, 6 samples were randomly numbered with 3 digits (453, 765, 987, 190, 286, 375), and the experimental results were still displayed in the form of the addition amount (0%, 2%, 4%, 6%, 8%, 10%) according to statistics [23].

The study was reviewed and approved by the United Nations Protection Force (UNPF) 989 Hospital, and informed consent was obtained from each subject prior to their participation in the study.

### 2.11. Statistical Analysis

All experiments were repeated at least three times, and the results were expressed as mean ± standard deviation. Microsoft Office Excel 2016 was used to summarize and tabulate the data. SPSS Statistics 26 software was used for a one–way analysis of variance. The Waller–Duncan multivariate range test was used to determine differences between samples (*p* < 0.05). Plots of experimental results were generated using Origin Pro 2022 software. Volatile compound information was processed using MassHunter Workstation B.07.00 software and compared to NIST14.

## 3. Results and Discussion

### 3.1. Effect of Microwave Treatment on the Basic Components of BSG

According to Table 1 below, BSGs have 25.62% protein (14.22% water–soluble protein), 18.31% dietary fiber (3.32% soluble dietary fiber), 13.52% starch, 7.13% fat, and 5.21% ash. In comparison, the protein content of MW–BSG showed an initial increase followed by a decrease with microwave treatment power, time, and material–liquid ratio. Fresh BSG has a considerable moisture content, making it vulnerable to microbial and spoilage bacteria. Microbial growth requires protein for nutrition; however, microwave heating eradicated the microorganisms and slowed down protein consumption in the BSGs. However, a higher microwave treatment power (>540 W) and time (>3 min) may potentially modify the structure and spatial conformations of proteins, resulting in their degradation [24]. The water–soluble protein–content increase can be ascribed to the improved thermal motion of protein molecules due to microwave treatment, the revelation of hydrophobic groups within the proteins, and the establishment of intermolecular hydrophobic conglomerates that lead to the formation of soluble protein aggregates [25]. The dietary fiber content of MW–BSG gradually decreased with microwave treatment power, time, and material–liquid ratio. This was because microwaves were able to penetrate lignocellulose, the main component of dietary fiber in BSGs, and caused a rapid internal heating, leading to fiber dissolution and fragmentation, ultimately resulting in the destruction of their original structure and dissolution [26]. The increase in soluble dietary fiber content due to the rapid increase in cell pressure of BSGs was caused by microwave heating. This resulted in the bursting of the cell wall and discharge of numerous active ingredients that assisted in the breakdown of insoluble dietary fiber into soluble dietary fiber [27]. The concentrations of starch, fat, and ash in MW–BSG increased significantly with treatment power, while time and material–liquid ratio increased. A high concentration of water–soluble protein and soluble dietary fiber have been found to be advantageous in bread processing and production [28]. However, the compositions of bread decreased when the material–liquid ratio exceeded 1:5. Therefore, the microwave treatment conditions of a 540 W power, 3 min, and a material–liquid ratio of 1:5 were chosen.

### 3.2. Effect of MW–BSG on the Specific Volume of Bread

**Figure 1:**
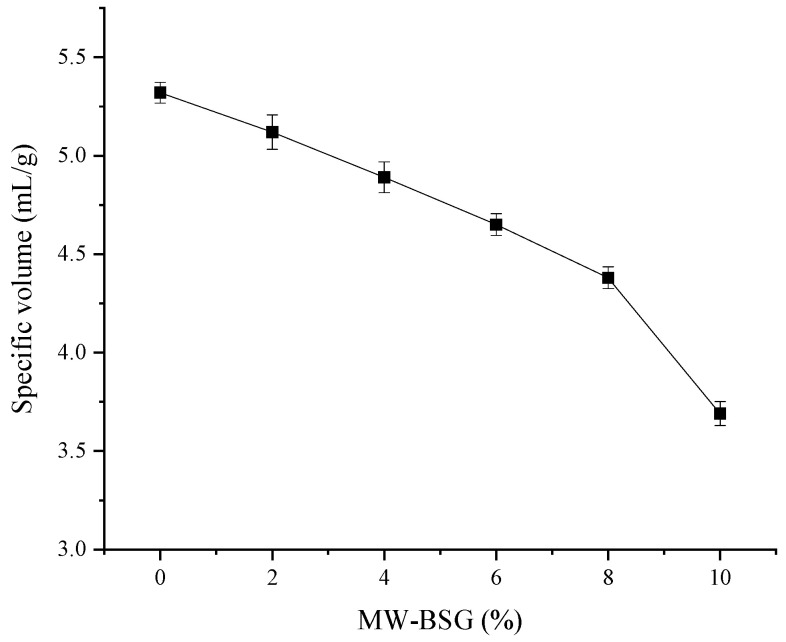
Effect of microwave–treated BSG (MW–BSG) on the specific volume of bread.

The specific volume of bread reflects the degree of expansion and the ability of the dough to retain its volume. It is generally accepted that the higher the specific volume, the better the quality of the bread. According to Figure 1, the specific volume of bread decreased as the amount of MW–BSG added increased, with a significant decrease observed when the amount exceeded 8%. Czubaszek et al. [4] demonstrated that the specific volume of bread decreased with increasing BSG content in wheat flour blends. The specific volume substantially decreased when the MW–BSG content exceeded 8%. This was because the microwave treatment increased the swelling capacity of BSG dietary fiber [29] and the contents of soluble dietary fiber and ash. Excessive soluble dietary fiber, such as β–glucan, can compete with wheat starch granules for moisture, limiting their swelling and pasting during bread baking [30]. Excessive ash and dietary fiber contents hindered gluten formation, affected the gluten network structure, reduced the dough’s ability to trap air during fermentation, and limited the post–expansion volume retention [31]. To achieve optimal results, it is essential not to exceed 8% addition of MW–BSG to bread.

### 3.3. Effect of MW–BSG on the Texture of Bread

It has been reported that there is a negative correlation between the quality of bread and its hardness, gumminess, and chewiness. If bread is harder, more adhesive, and chewier, its taste is worse. If bread is more elastic, cohesive, and resilient, its taste is better [32]. According to Table 2, the hardness, gumminess, and chewiness of bread first decreased and then increased, while the cohesiveness increased and then decreased; the springiness and resilience both decreased with the content of MW–BSG. This was because the addition of MW–BSG increased the content of dietary fiber in the bread, which had a certain water–absorbing capacity that prevented the formation of a gluten structure and reduced the extensibility of the dough, leading to a reduction in the springiness and resilience of the bread [30]. The increased soluble dietary fiber due to microwave treatment has a higher capacity for retaining water, which forms a network barrier to minimize water evaporation from the bread during baking [33]. This leads to a decrease in bread hardness, gumminess, and chewiness while increasing cohesiveness. When more than 6% of MW–BSG was added, the content of not only dietary fiber but also protein and starch increased. More proteins combined with water, forming a gel network, which reduced the pasting of starch granules [34]. This may alter the textural properties of bread, resulting in bread with higher hardness, gumminess, and chewiness, and lower cohesiveness.

### 3.4. Effect of MW–BSG on the Crust and Crumb Color of Bread

**Figure 2:**
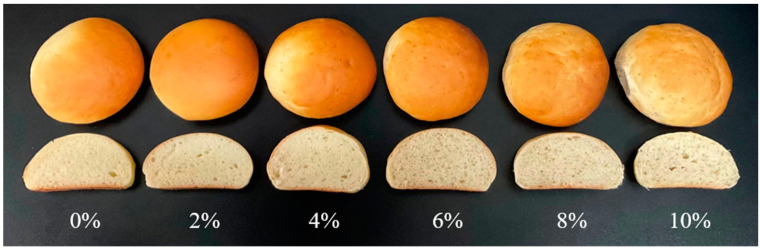
Pictures of bread with different MW–BSG additions. Note: 0%, 2%, 4%, 6%, 8%, and 10% indicate different MW–BSG additions, respectively.

Bread color is an important attribute that affects the preference of bread, with greater L* values indicating a brighter color, larger a* values (+) indicating a redder color, and greater b* values (+) indicating a more yellow color. According to Table 3, MW–BSG significantly changed the L*, a*, and b* values of the bread crust and crumb (*p* < 0.05). The L* value of the bread crust decreased and then increased; both the a* and b* values first increased and then decreased with the content of MW–BSG. While the L* and b* values of the crumb gradually decreased, the a* value generally increased with the content of MW–BSG. The total color difference ΔE is a comprehensive indicator of color change. The ΔE values for both the crust and crumb of the bread both exceed 1, indicating that consumers can observe the color change due to the addition of MW–BSG [35]. The color of the crust is primarily attributed to the Maillard reaction and can be easily affected by changes in temperature and moisture; the color of the crumb is predominantly determined by the color of the ingredients used [36]. MW–BSG contains high contents of proteins that facilitate the Maillard reaction [37], leading to the darkening, reddening, and yellowing of the bread crust’s color. The high phenolic content of BSG promote the formation of large brown molecules during bread baking [38]; combined with the brown hue of the BSG slurry, this darkens and reddens the bread crumb. Amoriello et al. [39] reported a gradual darkening of the bread crumb color with BSG, which is consistent with our results. The bread crust with BSG brightened in a previous study, whereas that with MW–BSG darkened in our study. Multiple variables may affect Maillard reaction, including temperature, moisture, pH, and the type of sugar utilized. The microwave treatment modified the physicochemical components of BSG, causing the insoluble dietary fiber to degrade into smaller molecules and increasing the content of soluble dietary fiber. This elevation in substrate content contributed to the Maillard reaction, resulting in a gradual darkening of the bread crust with the MW–BSG. However, surpassing 6% of added MW–BSG led to a lighter crust color, potentially due to the overall acidic pH of BSG as a co–product of beer fermentation. The accumulation of BSG influences bread’s pH, creating an acidic environment that hinders the Maillard reaction. Additionally, the inclusion of MW–BSG reduces the bread’s volume and increases the distance between the crust and the top of the oven, resulting in insufficient temperature during the crust–browning process and impeding the development of the crust color [40]. As shown in Figure 2, when the amount of MW–BSG added exceeds 6%, the crust of the bread gradually loses its attractive brown color and becomes uneven, while the crumb of the bread becomes darker. Therefore, it is recommended to control the addition amount of MW–BSG to within 6%.

### 3.5. Effect of MW–BSG on the Microstructure of Bread

**Figure 3:**
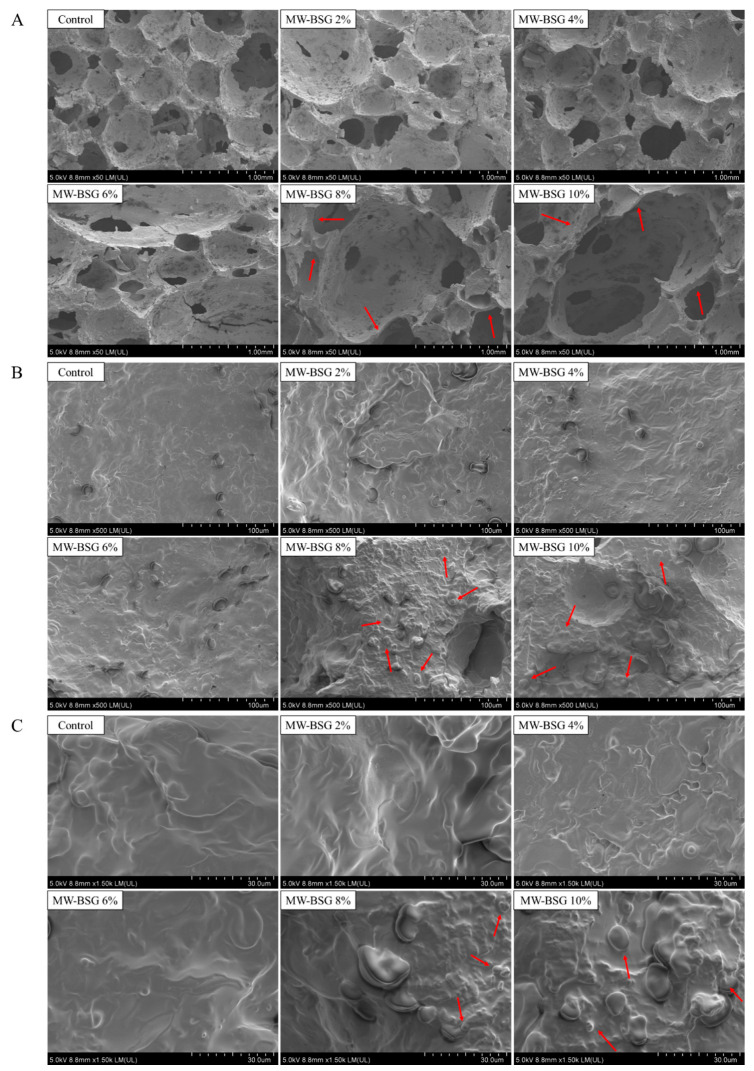
Scanning electron micrographs (SEM) of bread with different MW–BSG additions. (**A**) 50×, (**B**) 500×, (**C**) 1500×.

SEM was used to perform microstructural analysis at various magnifications, visually capturing the intrinsic structural data of the sample. Pore size and distribution are critical indicators of bread quality, with smaller pores and uniform distribution positively correlated with better–quality bread. Figure 3A indicates that the larger pores of bread with MW–BSG (>6%) became larger and distributed unevenly, which is mainly related to the inadequate gluten formation. More fiber, protein, and starch contents due to the addition of MW–BSG in the dough competed with gluten proteins for water, inhibiting the gluten formation. Microwave treatment notably raised the amounts of soluble dietary fiber and water–soluble protein, both of which possess a stronger hydration capacity compared to gluten proteins. Soluble fiber interacts non–covalently with gluten proteins via hydrogen bonding and hydrophobic interactions, whereas insoluble fiber disrupts the gluten network by competing with gluten proteins for water molecules [41]. Additionally, the limited ability of gluten to trap gas can result in the small bubbles either bursting and escaping into the air or merging with each other to form larger bubbles. This affects the amount of air pockets in the bread, leading to irregular sizes and uneven distribution. The number, size, and arrangement of pores in the bread are associated with its firmness and size. A decrease in the quantity and dimensions of stomata indicates the breakdown of the gluten network in the dough, resulting in reduced volume and inferior flexibility in bread formation, as described in Section 3.2 and Section 3.3. With the addition of 8% and 10% MW–BSG, the thicker stomatal pore walls of the bread were observed (Figure 3A). Some starch granules adhered to the surface of the gluten structure instead of being enclosed within the gluten network (Figure 3B), and the gluten matrix also showed unevenness and discontinuity (Figure 3C), all of which may have contributed to the increased bread hardness [42,43].

### 3.6. Effect of MW–BSG on the Flavor of Bread Crust and Crumb

#### 3.6.1. Electronic Nose

**Figure 4:**
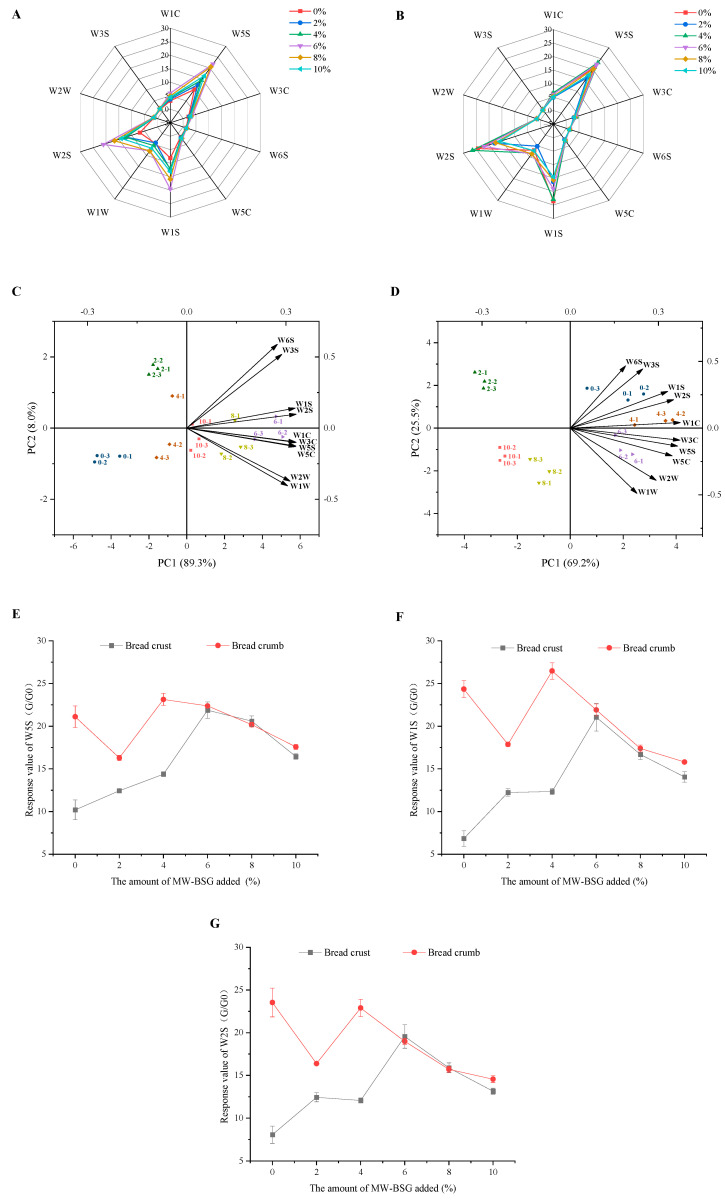
Radar plots of E–nose sensor response values for crust (**A**) and crumb (**B**) of bread with different MW–BSG contents, and biplane plots of E–nose sensor response values for crust (**C**) and crumb (**D**) of bread (different symbols and numbers represent different MW–BSG additions, and arrows indicate the effect of different sensors on the principal components); magnitude of E–nose sensor response values for bread crust and crumb for W5S (**E**), W1S (**F**), and W2S (**G**) sensors.

The E–nose is an aroma analysis device that effectively replicates the human sense of smell with the advantages of economy, portability, and ease of use. The PEN3 E–nose contains 10 metal sensors that capture diverse volatile organic compounds and provide data on the aroma intensity of the sample [44]. Based on Figure 4A,B, the response values of the E–nose sensors for the crust and crumb of the bread with MW–BSG were more distinct for three specific sensors, namely, W5S, W1S, and W2S. This indicates that MW–BSG has a greater effect on the potency of nitrogen oxides, methyl analogues, alcohols, and aldehydes and ketones aromas in bread flavor (Supplementary Material–Table S1). Fermentation and baking are critical elements that determine the aroma of bread, and the products of yeast fermentation and the Maillard reaction, such as alcohols, aldehydes, and ketones, are key contributors to the final aroma profile of bread [45]. The inclusion of MW–BSG has the potential to affect the Maillard reaction throughout the dough baking process. Figure 4E–G shows that MW–BSG leads to an increase in crust response values for the W5S, W1S, and W2S sensors, followed by a decrease. The crumb response values first decreased, followed by an increase, and then decreased. When the addition of MW–BSG was less than 6%, the response values of the crumb exceeded that of the crust. However, at the 6% addition point, the crust and crumb response values were essentially identical. Bread crust flavor is mainly derived from a thermal reaction during baking, whereas bread crumb flavor is generated by yeast enzymatic reaction during dough fermentation [46]. In Section 3.4, it is noted that adding more than 6% of MW–BSG could limit the occurrence of the Maillard reaction, which could cause the bread crust’s E–nose sensor response values to decrease. Also, the response value of the bread crumb’s E–nose sensor decreased when the MW–BSG addition was higher than 4%, possibly due to insufficient bread fermentation resulting from insufficient dough retention [47].

To explore the correlation between volatile compounds and samples, to identify the effect of different types of volatile flavor compounds on sample variation, and to verify the authenticity and reliability of the data, we performed principal component analysis (PCA) [48,49]. Based on Figure 4C,D, the combined contribution of principal component 1 (PC1) and principal component 2 (PC2) in the crust and crumb were 97.3% (PC1 89.3%, PC2 8.0%) and 94.7% (PC1 69.2%, PC2 25.5%), respectively, which represents the majority of the information in the raw data of the samples and ensures the authenticity of the results. No overlap was observed between any of the MW–BSG breads, and the control sample, indicating that there is significant variation in the aroma profile between samples [48]. This suggests that MW–BSG had a significant effect on the aroma of the bread. Based on the sensor loading analysis, it is clear that all the sensors contribute to PC1, while only some contribute to PC2. The sensors W1S and W2S have high contributions to both PC1 and PC2. These results suggested that the volatile flavor compounds responsible for discriminating MW–BSG breads from control breads were likely to be methyls, alcohols, aldehydes, and aromatic compounds [50]. For further investigation, an HS–SPME–GC–MS analysis was performed to identify the volatile compounds in MW–BSG bread.

#### 3.6.2. Determination of Volatile Compounds

**Figure 5:**
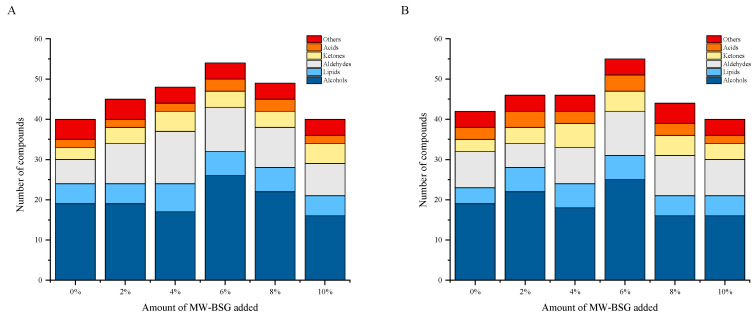
Number of different types of compounds in the crust (**A**) and crumb (**B**).

The GC–MS results of the crusts and crumbs of the breads are shown in Table 4 and Table 5, and the chromatograms are shown in Supplementary Material Figures S1 and S2. The results show that 77 flavor compounds were detected in the crusts, of which 40, 45, 48, 54, 49, and 40 compounds were detected at 0%, 2%, 4%, 6%, 8%, and 10% additions, respectively, which were mainly alcohols (34), lipids (17), aldehydes (9), ketones (7), and acids (4). A total of 72 compounds were detected in the bread crumbs: 42, 46, 46, 55, 44, and 40 compounds were detected at 0%, 2%, 4%, 6%, 8%, and 10% additions, respectively, consisting mainly of alcohols (32), lipids (15), aldehydes (8), ketones (8), and acids (4). These are all the major aroma compounds in bread reported in the literature [51,52]. The similarity of volatile compounds found in both crusts and crumbs of bread (Figure 5) corresponds to the results presented in Section 3.6.1, where both crusts and cores show similar response patterns across the ten sensors of the E–nose. Additionally, both the crust and the crumb of the breads contained high levels of methyls, alcohols, aldehydes, and aromatic compounds, such as ethanol, 3–methyl–1–butanol, 2–methyl–1–butanol, 2,3–butanediol, nonanal, furfural, acetic acid, phenylethyl alcohol, and limonene. Phenylethyl alcohol emits a rose and honey aroma, while limonene adds an orange and lemon aroma with pleasant characteristics, all of which contribute to the formation of the unique flavor of the bread.

Alcohols are the primary volatile flavor compounds in bread, mainly formed during the dough fermentation process, which gives bread a fruity and grassy aroma [53]. Alcohols present in bread crusts and crumbs accounted for 44.16% and 44.44% of the total volatile compound species, respectively, with ethanol and phenylethyl alcohol having the highest concentrations, accounting for over 90% of the total. MW–SBG resulted in an increase in the ethanol levels in both the bread crust and crumb. However, there was an initial increase and subsequent decrease in the total number of alcohol compounds with the content of BSG. The addition of BSG, a co–product of beer fermentation, to bread is known to increase the type and amount of volatile alcoholic compounds, such as ethanol, in the finished product. However, when the amount of BSG added exceeds 6%, there is a decrease in the type of alcoholic compounds. This may be due to the inhibitory effect of excess BSG on dough fermentation [54].

Differences in volatile compounds within bread are not only due to dough fermentation, but are also influenced by enzymatic reactions, lipid oxidation, and thermal reactions [55]. Esters have a sweet and fruity nature and can be produced through enzymatic processes [56]. The amount and composition of lipid volatiles in MW–BSG breads were higher than the control sample’s. The enzymatic reaction may be affected by the water content and distribution in the dough, while the high amount of insoluble dietary fiber in the BSG limits the activity of α–amylase by binding to the water. In this study, BSG was subjected to microwave pretreatment, which increased the soluble dietary fiber content and decreased the insoluble dietary fiber content. As a result, there was a redistribution of water in the dough and an increase in the formation of lipid volatiles.

Aldehyde volatiles have a fruity, nutty, and malty aroma and are formed during the oxidation of fats [57]. For example, the oxidation of linoleic acid produces (E)–2–nonenal and hexanal [58]. The aldehyde contents and composition in the breads showed a trend of an initial increase followed by a decrease with the content of MW–BSG. This can be attributed to two main factors. First, the presence of MW–BSG increases the lipid content of the bread, which serves as a substrate for the lipid reaction that produces the aldehyde compounds. Second, higher levels of MW–BSG incorporation reduce the dough’s ability to hold air, while insufficient oxygen contents inhibit lipid oxidation, resulting in reduced aldehyde production.

The decrease in acid volatiles in the MW–BSG breads compared to the control could be attributed to the inhibition of bread fermentation by MW–BSG. Acids were produced by yeast fermentation and subsequent aldehyde oxidation. Ketones were also affected by yeast fermentation. 2,3–Butanedione, an important aroma compound in bread, showed a positive correlation with the degree of bread fermentation and imparted a buttery and caramelized aroma to the bread [59]. However, none of these were detected in MW–BSG bread crusts. Furthermore, the Maillard reaction and caramelization reaction during bread baking produce 2–pentylfuran, furfural, and 3–furanmethanol as signature products [60]. However, when MW–BSG was added up to a concentration of 10%, the levels of all these volatiles decreased. This indicates that an excessive amount of MW–BSG could inhibit the Maillard reaction and the caramelization reaction. It is crucial to carefully control the addition of MW–BSG to avoid any negative effects on the reaction process.

### 3.7. Sensory Evaluation of BSG Bread

**Figure 6:**
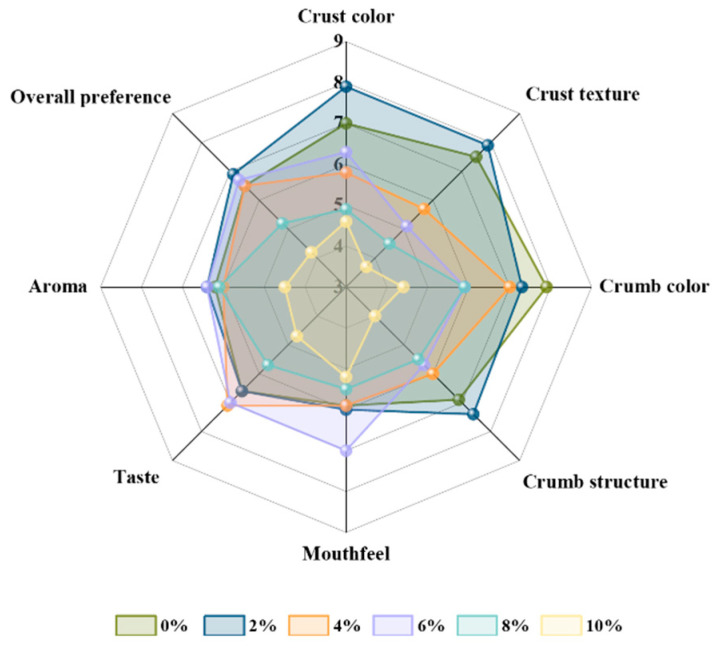
Radar chart of sensory evaluation of MW–BSG with different additions.

The overall sensory evaluation results show how consumers intuitively feel about MW–BSG bread, and how they comprehensively express their preferences for and acceptance of value–added products [61]. The sensory evaluation of the breads, as shown in Figure 6, showed more noticeable variations in crust color, crust texture, crumb color, and crumb structure due to the incorporation of MW–BSG. This is likely due to the nutrient–rich nature of BSG, which contains dietary fiber and proteins that affect dough fermentation and shaping, consequently affecting bread quality attributes such as texture, color, and volume. In addition, the microwave treatment resulted in an increase in soluble dietary fiber and water–soluble proteins, leading to a significant improvement in bread volume and color compared to the control group, consistent with our findings reported in Section 3.2, Section 3.3 and Section 3.4. While the control bread’s scores were generally higher, it received lower scores for crust color, crust texture, and crumb structure compared to the bread made with 2% MW–BSG. Mouthfeel scores were lower than those of the breads made with 2–6% MW–BSG, while taste scores were lower than those of the breads made with 4% and 6% MW–BSG. In addition, aroma scores were lower than those of the breads made with 2% and 6% MW–BSG. The overall preference scores were lower than those of the 2–6% MW–BSG breads. In summary, the sensory scores of the bread without BSG (mean score: 6.81 ± 0.84) were lower in the 2% MW–BSG breads (mean score: 7.05 ± 1.15). Meanwhile, the breads with 4% and 6% MW–BSG (mean scores: 6.25 ± 0.44 and 6.26 ± 0.45) had scores closer to the control, while the breads with 8% and 10% MW–BSG (mean scores: 5.41 ± 0.93 and 4.41 ± 0.78) had lower scores. Therefore, our recommendation is that the addition of up to 6% MW–BSG to bread is acceptable based on consumer preference.

## 4. Conclusions

This study showed that microwave–treated BSG had a significant effect on the quality and flavor characteristics of bread. Microwave treatment effectively improved the soluble dietary fiber and water–soluble proteins content of BSG, which, in turn, helped counteract the negative effects of BSG on bread quality. The incorporation of MW–BSG into bread reduced the specific volume of the bread. However, the texture, color, flavor, and microstructure of the bread were not adversely affected by the addition of 0–6% MW–BSG. Importantly, the addition of 6% MW–BSG to the bread resulted in an increase in the content of aroma compounds. This resulted in a more intense flavor. In addition, sensory evaluations showed that consumers found the addition of up to 6% MW–BSG acceptable. Overall, the study showed that bread can tolerate the addition of up to 6% MW–BSG. Our findings provide potential avenues for the research and utilization of beer co–products. Further research is needed to analyze the functional properties of MW–BSG bread, including digestibility, and to conduct sensory validations in different countries and populations so that the bread can be more effectively marketed for consumption.

## Figures and Tables

**Table 1:** Effect of microwave treatment on the main components of BSG (%).

Components		Protein (Water–Soluble Protein)	Dietary Fiber (Soluble Dietary Fiber)	Starch	Fat	Ash
Power (W) (3 min; 1:5 material–liquid ratio)	0	25.62 ± 0.25 ^c^ (14.22 ± 0.35 ^d^)	18.31 ± 0.16 ^a^ (3.32 ± 0.25 ^d^)	13.52 ± 0.11 ^d^	7.13 ± 0.19 ^c^	5.21 ± 0.02 ^d^
	360	25.65 ± 0.37 ^c^ (15.60 ± 0.24 ^c^)	18.16 ± 0.18 ^b^ (4.56 ± 0.23 ^c^)	13.86 ± 0.05 ^c^	7.69 ± 0.07 ^a^	5.33 ± 0.01 ^c^
	540	26.12 ± 0.29 ^a^ (17.54 ± 0.16 ^a^)	17.73 ± 0.22 ^c^ (8.13 ± 0.09 ^a^)	15.12 ± 0.28 ^a^	7.38 ± 0.09 ^b^	5.41 ± 0.03 ^b^
	720	25.92 ± 0.58 ^b^ (16.86 ± 0.23 ^b^)	17.02 ± 0.18 ^d^ (6.33 ± 0.20 ^b^)	15.04 ± 0.35 ^ab^	7.69 ± 0.05 ^a^	5.56 ± 0.05 ^a^
Time (min) (540 W; 1:5 material–liquid ratio)	0	25.62 ± 0.17 ^bc^ (14.22 ± 0.12 ^c^)	18.31 ± 0.12 ^a^ (3.32 ± 0.17 ^d^)	13.52 ± 0.26 ^d^	7.13 ± 0.20 ^c^	5.21 ± 0.22 ^c^
	1	25.64 ± 0.13 ^bc^ (15.73 ± 0.22 ^b^)	18.24 ± 0.27 ^a^ (5.44 ± 0.21 ^b^)	14.35 ± 0.18 ^c^	7.34 ± 0.19 ^ab^	5.55 ± 0.18 ^b^
	3	26.12 ± 0.27 ^a^ (17.54 ± 0.10 ^a^)	17.73 ± 0.16 ^b^ (8.13 ± 0.26 ^a^)	15.12 ± 0.17 ^a^	7.38 ± 0.17 ^ab^	5.41 ± 0.16 ^b^
	5	25.88 ± 0.31 ^b^ (17.64 ± 0.29 ^a^)	17.59 ± 0.14 ^c^ (5.09 ± 0.31 ^c^)	14.68 ± 0.16 ^b^	7.47 ± 0.25 ^a^	5.71 ± 0.13 ^a^
Material–liquid ratio (3 min; 540 W)	0	25.62 ± 0.17 ^b^ (14.22 ± 0.23 ^d^)	18.31 ± 0.19 ^a^ (3.32 ± 0.28 ^d^)	13.52 ± 0.22 ^d^	7.13 ± 0.25 ^d^	5.21 ± 0.27 ^d^
	1:3	26.22 ± 0.29 ^a^ (16.01 ± 0.23 ^bc^)	17.94 ± 0.10 ^b^ (4.67 ± 0.07 ^c^)	14.78 ± 0.12 ^c^	7.51 ± 0.12 ^b^	5.65 ± 0.15 ^a^
	1:5	26.12 ± 0.22 ^a^ (17.54 ± 0.13 ^a^)	17.73 ± 0.26 ^b^ (8.13 ± 0.14 ^a^)	15.12 ± 0.15 ^b^	7.38 ± 0.27 ^c^	5.41 ± 0.28 ^b^
	**1:7**	25.73 ± 0.26 ^b^ (16.29 ± 0.12 ^b^)	16.78 ± 0.27 ^c^ (7.15 ± 0.08 ^b^)	15.57 ± 0.07 ^a^	7.88 ± 0.15 ^a^	5.33 ± 0.17 ^c^

Note: Results are expressed as mean ± standard deviation (n = 3), and different letters in the same column indicate a significant difference (*p* < 0.05).

**Table 2:** Effect of MW–BSG on the texture of bread.

MW–BSG/%	Hardness/N	Springiness	Cohesiveness/N	Gumminess	Chewiness	Resilience
0	578.12 ± 41.12 ^c^	0.89 ± 0.06 ^a^	0.59 ± 0.01 ^c^	338.25 ± 23.45 ^c^	298.14 ± 12.15 ^b^	0.24 ± 0.01 ^a^
2	558.65 ± 34.67 ^d^	0.85 ± 0.03 ^a^	0.62 ± 0.02 ^b^	329.71 ± 13.23 ^d^	268.92 ± 13.22 ^c^	0.25 ± 0.01 ^a^
4	524.15 ± 30.22 ^e^	0.76 ± 0.01 ^b^	0.63 ± 0.01 ^b^	314.11 ± 12.21 ^e^	245.39 ± 11.15 ^d^	0.23 ± 0.01 ^a^
6	501.65 ± 25.26 ^f^	0.72 ± 0.04 ^bc^	0.66 ± 0.02 ^a^	302.35 ± 10.45 ^f^	235.63 ± 9.21 ^e^	0.22 ± 0.01 ^b^
8	601.65 ± 21.72 ^b^	0.71 ± 0.02 ^bc^	0.62 ± 0.03 ^b^	358.92 ± 11.08 ^b^	291.37 ± 15.11 ^b^	0.20 ± 0.01 ^b^
10	1168.62 ± 35.22 ^a^	0.62 ± 0.03 ^d^	0.55 ± 0.01 ^d^	498.64 ± 33.45 ^a^	362.25 ± 16.09 ^a^	0.16 ± 0.01 ^c^

Note: Results are expressed as mean ± standard deviation (n = 3), and different letters in the same column indicate a significant difference (*p* < 0.05).

**Table 3:** Effect of MW–BSG on the color of bread crust and crumb.

MW–BSG/%	Bread Crust				Bread Crumb			
	L*	a*	b*	ΔE	L*	a*	b*	ΔE
0	55.52 ± 0.81 ^c^	14.23 ± 0.23 ^b^	30.29 ± 0.65 ^a^	—	66.97 ± 0.16 ^a^	1.66 ± 0.05 ^d^	16.07 ± 0.14 ^a^	—
2	53.18 ± 0.23 ^d^	14.84 ± 0.11 ^ab^	28.79 ± 0.07 ^b^	2.85 ± 0.18 ^bc^	63.61 ± 0.33 ^b^	1.90 ± 0.02 ^c^	15.22 ± 0.16 ^bc^	3.47 ± 0.33 ^d^
4	52.55 ± 0.31 ^d^	14.58 ± 0.25 ^b^	28.6 ± 0.43 ^b^	3.44 ± 0.5 ^b^	61.75 ± 0.03 ^c^	2.45 ± 0.01 ^ab^	15.79 ± 0.17 ^ab^	5.29 ± 0.04 ^c^
6	51.33 ± 0.45 ^e^	15.26 ± 0.21 ^a^	28.61 ± 0.03 ^b^	4.63 ± 0.46 ^a^	61.28 ± 0.27 ^c^	2.52 ± 0.04 ^ab^	15.10 ± 0.50 ^c^	5.85 ± 0.25 ^b^
8	57.24 ± 0.40 ^b^	12.49 ± 0.64 ^c^	29.50 ± 0.60 ^ab^	2.68 ± 0.3 ^c^	60.58 ± 0.31 ^d^	2.35 ± 0.18 ^b^	13.86 ± 0.47 ^d^	6.81 ± 0.35 ^a^
10	58.78 ± 0.61 ^a^	11.25 ± 0.29 ^d^	28.88 ± 0.58 ^b^	4.69 ± 0.16 ^a^	59.98 ± 0.23 ^e^	2.56 ± 0.13 ^a^	14.79 ± 0.22 ^c^	7.17 ± 0.23 ^a^

Note: Results are expressed as mean ± standard deviation (n = 3); different letters are marked in the same column to indicate different significance levels (*p* < 0.05); the L*, a*, b* values of 0% bread crust and 0% bread wick are used as standard reference values to calculate ΔE, and the calculation formula is Equation (2).

**Table 4:** Identification of volatile compounds in bread crust.

No.	Compounds	CAS	Compound Concentration (ng/g)					
			0%	2%	4%	6%	8%	10%
	**Alcohols**							
1	1–isobutoxy–2–propanol	23436–19–3	–	–	2.70 ± 1.28 ^a^	1.02 ± 0.43 ^a^	–	–
2	Ethanol	64–17–5	3877.55 ± 13.03 ^b^	2340.67 ± 168.74 ^d^	1764.67 ± 293.55 ^e^	2918.36 ± 392.85 ^c^	3486.38 ± 328.71 ^c^	5045.22 ± 866.31 ^a^
3	(1E)–buta–1,3–dien–1–ol	70411–98–2	135.49 ± 1.83 ^a^	59.98 ± 11.61 ^b^	–	50 ± 8.81 ^b^	–	–
4	3–Butanolal	107–89–1	–	–	–	5.43 ± 1.54	–	–
5	2–Hexadecanol	14852–31–4	7.34 ± 1.64 ^a^	–	0.49 ± 0.23 ^d^	2.37 ± 1.52 ^c^	2.46 ± 1.23 ^bc^	3.37 ± 0.94 ^b^
6	2–Methyl–1–propanol	78–83–1	17.26 ± 12.34 ^d^	61.25 ± 4.61 ^b^	59.29 ± 1.62 ^b^	94.52 ± 10.28 ^a^	99.24 ± 1.15 ^a^	36.28 ± 11.93 ^c^
7	5–ethyl–2–heptanol	19780–40–6	–	–	–	1.07 ± 0.35 ^a^	0.83 ± 0.02 ^a^	–
8	3–Hexanol	623–37–0	–	–	–	8.02 ± 3.7 ^b^	6.56 ± 3.66 ^b^	30.78 ± 0.2 ^a^
9	2–Methylbutan–1–ol	137–32–6	174.37 ± 44.33 ^a^	88.26 ± 1.15 ^b^	82.62 ± 1.98 ^b^	140.56 ± 6.36 ^a^	148.81 ± 7.19 ^a^	155.1 ± 35.13 ^a^
10	3–Methyl–1–butanol	123–51–3	391.3 ± 18.18 ^a^	251.49 ± 2.91 ^b^	251.76 ± 19.17 ^b^	412.64 ± 21.16 ^a^	387.13 ± 33.47 ^a^	460.32 ± 87.56 ^a^
11	2–Hexyl–1–decanol	2425–77–6	–	4.7 ± 3.91	–	–	–	–
12	1–Octanol,2–butyl–	3913–02–8	–	11.1 ± 3.69 ^a^	–	1.37 ± 0.32 ^b^	–	–
13	Pentanol	71–41–0	32.76 ± 2.93 ^a^	14.15 ± 0.81 ^b^	8.82 ± 2.85 ^c^	19.95 ± 5.62 ^b^	12.58 ± 3.21 ^bc^	39.87 ± 9.41 ^a^
14	(E)–para–2,8–1–Menthadienol	7212–40–0	–	13.38 ± 5.94 ^a^	–	8.54 ± 1.04 ^a^	10.11 ± 2.78 ^a^	–
15	Benzoic acid, hex–2–en–1–ol	76841–70–8	8.73 ± 1.24	–	–	–	–	–
16	6,10,14–trimethyl–2–Pentadecanol	69729–17–5	–	3.91 ± 0.74 ^a^	–	2.77 ± 0.51 ^a^	2.52 ± 2.15 ^a^	–
17	1–Hexanol	111–27–3	78.72 ± 19.27 ^ab^	53.84 ± 1.6 ^b^	40.55 ± 13.18 ^b^	48.29 ± 9.91 ^b^	56.74 ± 5.17 ^b^	94.94 ± 5.59 ^a^
18	2–Heptanol, 3–methyl–	31367–46–1	2.29 ± 0.17	–	–	–	–	–
19	(2R,3R)–(–)–2,3–Butanediol	24347–58–8	247.85 ± 136.78 ^a^	76.73 ± 9.23 ^b^	50.48 ± 7.31 ^c^	70.82 ± 6.02 ^b^	115.78 ± 16.86 ^a^	53.8 ± 9.88 ^c^
20	Linalool	78–70–6	–	4.6 ± 1.91 ^b^	2.51 ± 0.74 ^b^	3.75 ± 1.32 ^b^	2.51 ± 1.56 ^b^	11.71 ± 2.27 ^a^
21	1–Octanol	111–87–5	11.59 ± 0.05 ^a^	–	4.75 ± 1.24 ^c^	7.1 ± 1.93 ^bc^	9.82 ± 3.08 ^ab^	12.9 ± 2.28 ^a^
22	(R)–(–)–3–METHYL–2–BUTANOL	1572–93–6	4.74 ± 2.02 ^a^	–	2.23 ± 2.2 ^ab^	1.37 ± 0.38 ^b^	2.8 ± 0.11 ^a^	–
23	He xylene glycol	5683–44–3	2.98 ± 0.09	–	–	–	–	–
24	(Z)–2–octen–1–ol	26001–58–1	4.6 ± 2.08 ^ab^	–	–	3.37 ± 1.19 ^b^	2.6 ± 0.08 ^b^	5.55 ± 0.99 ^a^
25	3–Furancarbinol	4412–91–3	–	37.21 ± 17.74 ^a^	10.31 ± 1.24 ^c^	16.24 ± 2.84 ^b^	22 ± 9.59 ^ab^	–
26	Furfuryl alcohol	98–00–0	50.55 ± 1.44 ^a^	–	–	–	–	24.14 ± 6.16 ^b^
27	Sorbitol	50–70–4	–	–	14.69 ± 8.7 ^ab^	22.81 ± 2.37 ^a^	14.33 ± 10.33 ^ab^	9.77 ± 7.19 ^b^
28	1,2,6–Hexanetriol	106–69–4	–	15.25 ± 2.83 ^a^	–	10.31 ± 1.07 ^b^	11.9 ± 1.11 ^ab^	–
29	1–deoxy–D–mannitol	60965–81–3	–	–	0.31 ± 0.25 ^a^	0.48 ± 0.28 ^a^	0.58 ± 0.25 ^a^	–
30	Longiborneol	465–24–7	–	12.24 ± 1.41	–	–	–	–
31	5–Methyl–2–furanmethanol	3857–25–8	4.19 ± 0.8	–	–	–	–	–
32	Hexahydro farnesol	6750–34–1	–	21.97 ± 23.97	–	–	–	–
33	Phenylethyl alcohol	60–12–8	568.09 ± 225.46 ^bc^	580.32 ± 377.39 ^abc^	213.67 ± 20.42 ^d^	344.25 ± 56.97 ^c^	518.08 ± 63.18 ^b^	852.74 ± 35.47 ^a^
34	OCTOXYNOL–5	2315–64–2	14.42 ± 0.83 ^c^	35.76 ± 0.42 ^a^	16.12 ± 2.36 ^c^	21.1 ± 0.65 ^b^	21.9 ± 11.56 ^bc^	20.66 ± 2.02 ^b^
	**Aldehydes**							
35	Isovaleraldehyde	590–86–3	–	–	1.23 ± 0.25	–	–	–
36	Hexanal	66–25–1	18.68 ± 4.42 ^ab^	14.88 ± 0.59 ^b^	12.92 ± 0.06 ^c^	20.87 ± 2.91 ^a^	–	16.24 ± 1.6 ^b^
37	(Z)–7–Hexadecenal	56797–40–1	–	–	2.43 ± 0.73 ^ab^	2.76 ± 0.02 ^b^	5.28 ± 2.4 ^a^	–
38	Nonanal	124–19–6	102.18 ± 27.32 ^ab^	81.88 ± 24.36 ^abc^	31.93 ± 7.5 ^d^	56.89 ± 8.31 ^bc^	62.31 ± 11.16 ^bc^	135.35 ± 30.14 ^a^
39	Furfural	98–01–1	100.72 ± 6.69 ^b^	267.25 ± 9.34 ^a^	38.56 ± 4.28 ^d^	65.45 ± 10.16 ^c^	76.53 ± 14.86 ^c^	15.11 ± 5.19 ^e^
40	7–Hydroxy–3,7–dimethyloctanal	107–75–5	–	5.53 ± 3.39	–	–	–	–
41	Benzaldehyde	100–52–7	178.88 ± 27.07 ^b^	612.36 ± 64.06 ^a^	68.87 ± 9.23 ^c^	138.1 ± 27.94 ^b^	160.59 ± 5.12 ^b^	178.35 ± 50.36 ^b^
42	(2E)–2–Nonenal	18829–56–6	11.18 ± 2.62 ^a^	–	5.76 ± 1.09 ^b^	–	5.67 ± 1.55 ^b^	11.36 ± 2.69 ^a^
43	Octanal	124–13–0	–	–	–	0.62 ± 0.11 ^a^	0.73 ± 0.25 ^a^	–
	**Lipids**							
44	Ethyl acetate	141–78–6	–	–	1.6 ± 0.35 ^b^	–	–	44.65 ± 32.01 ^a^
45	Vinyl acetate	108–05–4	–	36.15 ± 0.38 ^c^	26.6 ± 1.72 ^d^	40.94 ± 12.54 ^bc^	50.16 ± 6.54 ^b^	93.97 ± 10.93 ^a^
46	Ethyl butanoate	105–54–4	–	–	–	–	2.16 ± 0.05 ^b^	5.53 ± 0.17 ^a^
47	Arachic acid benzyl ester	77509–04–7	–	6.97 ± 2.71 ^a^	6.51 ± 2.99 ^a^	5.93 ± 0.26 ^a^	–	–
48	12,15–Octadecadiynoic acid methyl ester	57156–95–3	16.19 ± 1.71 ^a^	8.91 ± 1.39 ^b^	6.98 ± 0.22 ^c^	6.54 ± 1.49 ^bc^	–	–
49	Ethyl hexanoate	123–66–0	16.37 ± 3.15 ^a^	3.47 ± 1.71 ^d^	5.83 ± 2.76 ^cd^	8.51 ± 1.91 ^c^	12.5 ± 0.7 ^b^	20.82 ± 5.51 ^a^
50	Benzylcarbinyl caproate	6290–37–5	–	7.62 ± 0.72 ^a^	5.11 ± 1.1 ^b^	6.35 ± 2.64 ^ab^	7.24 ± 1.8 ^ab^	–
51	Docosyl docosanoate	17671–27–1	–	–	1.27 ± 0.78	–	–	–
52	Dodecan–2–yl 2,2,2–trifluoroacetate	1894–68–4	–	–	1.22 ± 0.04 ^a^	0.87 ± 0.03 ^b^	0.47 ± 0.05 ^c^	–
53	Ethyl caprylate	106–32–1	74.62 ± 14.28 ^ab^	–	32.21 ± 2.66 ^c^	54.02 ± 7.52 ^b^	81.84 ± 10.77 ^a^	100.73 ± 17.01 ^a^
54	Gamma–Butyrolactone	96–48–0	10.87 ± 2.56	–	–	–	–	–
55	Ethyl caprate	110–38–3	8.2 ± 2.0 ^bc^	5.77 ± 0.44 ^c^	3.21 ± 0.47 ^d^	6.27 ± 0.32 ^c^	10.81 ± 1.56 ^ab^	14.12 ± 2.35 ^a^
56	Acetic acid, 2–chloro–, nonyl ester	5451–96–7	–	–	0.54 ± 0.26 ^b^	0.57 ± 0.5 ^ab^	3.35 ± 2.3 ^a^	–
57	Geranyl isovalerate	109–20–6	–	38.14 ± 7.78	–	–	–	–
58	Allyl 2–ethylbutyrate	7493–69–8	12.89 ± 2.66 ^bc^	27.53 ± 13.84 ^ab^	5.26 ± 0.98 ^d^	17.88 ± 7.28 ^ab^	11.76 ± 1.14 ^c^	20.13 ± 2.01 ^a^
59	3–hydroxy Stearic Acid methyl ester	2420–36–2	–	15.09 ± 3.54	–	–	–	–
60	Ethyl laurate	106–33–2	–	7.58 ± 3.72 ^a^	–	1.87 ± 0.04 ^c^	3.96 ± 1.75 ^a^	6.39 ± 1.08 ^a^
	**Ketones**							
61	Butane–2,3–dione	431–03–8	54.05 ± 2.1	–	–	–	–	–
62	2–Heptanone	110–43–0	–	20.65 ± 7.04 ^ab^	13 ± 10.1 ^bc^	5.7 ± 0.7 ^c^	13.09 ± 6.02 ^b^	30.45 ± 4.35 ^a^
63	Hydroxy Propanone	116–09–6	–	–	15.04 ± 4.72 ^b^	28.79 ± 2.68 ^a^	–	19.46 ± 1.11 ^b^
64	2–Nonanone	821–55–6	33.52 ± 5.21 ^a^	26.28 ± 9.34 ^ab^	12.23 ± 1.17 ^c^	–	25.29 ± 3.21 ^ab^	20.71 ± 1.78 ^b^
65	2–Undecanone	112–12–9	13.48 ± 3.38 ^b^	19.31 ± 1.63 ^a^	8.06 ± 0.29 ^c^	11.79 ± 0.65 ^b^	14.54 ± 1.07 ^b^	9.81 ± 4.65 ^bc^
66	Acetophenone	98–86–2	–	100.35 ± 19.09	–	–	–	–
67	1–(3,5–di–tert–butyl–4–hydroxy–phenyl)–propan–1–one	14035–34–8	–	–	10.39 ± 1.03 ^c^	14.74 ± 0.86 ^b^	16.94 ± 7.51 ^abc^	25.55 ± 3.32 ^a^
	**Acids**							
68	2–hydroxydecanoic acid	5393–81–7	0.82 ± 0.06	–	–	–	–	–
69	Acetic acid	64–19–7	558.12 ± 66.07 ^a^	335.42 ± 47.25 ^c^	221.32 ± 8.17 ^d^	355.64 ± 48.08 ^c^	460.01 ± 0.28 ^b^	195.09 ± 40.9 ^d^
70	Isobutyric acid	79–31–2	–	–	11.92 ± 5.37 ^a^	2.3 ± 0.2 ^c^	3.6 ± 2.09 ^b^	–
71	4–hydroxybutyric acid	591–81–1	–	6.91 ± 0.64 ^b^	5.7 ± 0.4 ^c^	8.27 ± 1.87 ^ab^	9.74 ± 1.11 ^a^	10.75 ± 2.02 ^a^
	**Others**							
72	Limonene	138–86–3	88.89 ± 15.7 ^a^	51.91 ± 0.05 ^c^	51.5 ± 11.78 ^c^	67.24 ± 34.95 ^abc^	53.42 ± 17.1 ^bc^	78.87 ± 15.51 ^ab^
73	2–Amylfuran	3777–69–3	19.09 ± 6.68 ^a^	14.56 ± 5.56 ^abc^	5.92 ± 2.55 ^d^	8.49 ± 1.24 ^cd^	11.03 ± 0.69 ^b^	29.2 ± 17 ^a^
74	Acetoin	513–86–0	1335.32 ± 333.77 ^a^	686.56 ± 49.69 ^c^	429.04 ± 27.41 ^d^	686.4 ± 92.72 ^bc^	789.65 ± 113.16 ^bc^	769.05 ± 30.8 ^b^
75	Tetramethylpyrazine	1124–11–4	–	9.37 ± 5.25	–	–	–	–
76	2–Acetylthiazole	24295–03–2	6.94 ± 1.81 ^a^	3.6 ± 0.71 ^b^	2.33 ± 0.5 ^c^	2.08 ± 0.42 ^c^	3.94 ± 0.71 ^b^	5.47 ± 1.19 ^ab^
77	2,4–DI–TERT–BUTYLTHIOPHENOL	19728–43–9	1.43 ± 1.0	–	–	–	–	–

Note: “–” means not detected. Results are expressed as mean ± standard deviation (n = 3); different letters are marked in the same row to indicate different significance levels (*p* < 0.05).

**Table 5:** Identification of volatile compounds in bread crumb.

No.	Compounds	CAS	Compound Concentration (ng/g)					
			0%	2%	4%	6%	8%	10%
	**Alcohols**							
1	Ethanol	64–17–5	2542.34 ± 412.83 ^cd^	2303.04 ± 0.16 ^d^	2776.1 ± 78.59 ^c^	3616.8 ± 162.38 ^b^	4704.09 ± 67.43 ^a^	4448.21 ± 370.1 ^a^
2	(1E)–buta–1,3–dien–1–ol	70411–98–2	40.9 ± 12.02 ^b^	–	–	136.89 ± 30.55 ^a^	–	–
3	2–Pentanol, 3–ethyl–	609–27–8	–	–	–	3.03 ± 1.5	–	–
4	3–Butanolal	107–89–1	–	–	–	3.96 ± 1.41	–	–
5	2–Methyl–1–propanol	78–83–1	83.75 ± 14.37 ^ab^	53.3 ± 1.13 ^c^	75.78 ± 1.04 ^b^	100.84 ± 1.17 ^a^	42.11 ± 4.63 ^d^	49.12 ± 13.87 ^cd^
6	5–ethyl–2–heptanol	19780–40–6	–	–	–	0.9 ± 0.23	–	–
7	2–Hexadecanol	14852–31–4	–	1.4 ± 0.23 ^a^	1.03 ± 0.1 ^b^	0.95 ± 0.04 ^b^	–	–
8	α,β–dimethyl–Benzeneethanol	52089–32–4	–	–	–	35.9 ± 6.38	–	–
9	3–Hexanol	623–37–0	–	–	–	–	–	50.27 ± 6.57
10	2–Methylbutan–1–ol	137–32–6	–	88.25 ± 12.15 ^c^	100.01 ± 4.17 ^c^	146.67 ± 9.61 ^b^	173.77 ± 11.61 ^a^	171.73 ± 33.35 ^ab^
11	3–Methyl–1–butanol	123–51–3	308.18 ± 5.32 ^c^	232.89 ± 15.49 ^d^	353.04 ± 13.61 ^b^	427.19 ± 29.85 ^a^	442.9 ± 17.4 ^a^	490.8 ± 68.79 ^a^
12	2–hexyloctan–1–ol	19780–79–1	1.33 ± 1.01 ^a^	0.86 ± 0.42 ^a^	–	–	–	–
13	Pentanol	71–41–0	11.89 ± 1.6 ^cd^	11.25 ± 0.51 ^d^	13.02 ± 1.15 ^c^	26.42 ± 0.97 ^b^	–	36.18 ± 8.35 ^a^
14	(E)–para–2,8–1–menthadienol	7212–40–0	9.79 ± 3.08 ^a^	–	–	12.33 ± 0.99 ^a^	–	–
15	6,10,14–trimethyl–2–Pentadecanol	69729–17–5	2.51 ± 0.57 ^ab^	2.12 ± 0.32 ^b^	2.85 ± 0.23 ^a^	–	–	–
16	1–Hexanol	111–27–3	46.65 ± 11.74 ^c^	57.71 ± 1.41 ^c^	55.84 ± 4.09 ^c^	78.95 ± 6.53 ^b^	86.43 ± 11.76 ^ab^	103.41 ± 10.99 ^a^
17	4–Methoxymethoxy–2–ethyl–2–butanol	37587–78–3	–	–	–	1.62 ± 0.24	–	–
18	(Z)–2–nonen–1–ol	41453–56–9	–	1.7 ± 0.03	–	–	–	–
19	1–Octen–3–ol	3391–86–4	–	–	–	–	–	27.29 ± 4.13
20	Linalool	78–70–6	1.54 ± 0.02 ^d^	4.4 ± 1.09 ^b^	4.79 ± 0.13 ^b^	6.21 ± 1.32 ^b^	1.06 ± 0.21 ^c^	12.33 ± 1.67 ^a^
21	1–Octanol	111–87–5	7.21 ± 1.14 ^cd^	7.16 ± 0.76 ^d^	8.51 ± 0.42 ^c^	11.36 ± 0.61 ^b^	15.82 ± 6.64 ^ab^	14.09 ± 0.7 ^a^
22	(2R,3R)–(–)–2,3–Butanediol	513–85–9	11.61 ± 3 ^cd^	10 ± 1.57 ^d^	13.83 ± 0.03 ^c^	29.05 ± 4.32 ^b^	43.3 ± 4.64 ^a^	15.79 ± 7.93 ^cd^
23	(R)–(–)–3–METHYL–2–BUTANOL	1572–93–6	–	1.71 ± 0.21 ^b^	2.98 ± 1.09 ^ab^	3.89 ± 1.41 ^a^	3.96 ± 1.8 ^a^	–
24	(Z)–2–octen–1–ol	26001–58–1	–	2.75 ± 0.86 ^c^	4.1 ± 0.65 ^c^	5.85 ± 0.89 ^ab^	5.41 ± 0.1 ^b^	7.52 ± 0.99 ^a^
25	3–Furancarbinol	4412–91–3	28.23 ± 2.57 ^b^	18.26 ± 2.39 ^c^	18.66 ± 0.51 ^c^	27.37 ± 4.96 ^b^	47.67 ± 7.1 ^a^	–
26	Furfuryl alcohol	98–00–0	–	–	–	–	–	18.17 ± 1.11
27	Sorbitol	50–70–4	5.2 ± 2.34 ^b^	21.91 ± 0.71 ^a^	25.11 ± 9.11 ^a^	20.27 ± 6.26 ^a^	22.18 ± 8.2 ^a^	2.68 ± 0.72 ^b^
28	1,2,6–Hexanetriol	106–69–4	6.82 ± 1.41 ^c^	8.97 ± 0.46 ^b^	–	12.8 ± 2.61 ^a^	12.67 ± 7.64 ^abc^	–
29	1–deoxy–D–mannitol	60965–81–3	0.31 ± 0.12 ^d^	0.76 ± 0.17 ^c^	1.32 ± 0.02 ^b^	2.35 ± 0.31 ^a^	1.11 ± 0.5 ^bc^	–
30	5–Methyl–2–furanmethanol	3857–25–8	2.95 ± 0.7 ^a^	3.09 ± 0.6 ^a^	–	–	–	–
31	Phenylethyl alcohol	60–12–8	303.51 ± 38.74 ^de^	281.38 ± 10.33 ^e^	371.4 ± 40.65 ^d^	534.77 ± 77.83 ^c^	775.54 ± 39.74 ^b^	1051.14 ± 56.08 ^a^
32	OCTOXYNOL–5	2315–64–2	4.82 ± 0.19 ^e^	6.04 ± 0.36 ^d^	16.44 ± 5.47 ^c^	27.44 ± 1.78 ^b^	67.49 ± 17.51 ^a^	20.09 ± 2.84 ^c^
	**Aldehydes**							
33	Crotonaldehyde	123–73–9	–	96.66 ± 19.97	–	–	–	–
34	Hexanal	66–25–1	14.6 ± 3.93 ^ab^	11.62 ± 1.42 ^b^	13.57 ± 1.08 ^b^	21.78 ± 5.05 ^a^	–	6.68 ± 2.92 ^c^
35	(Z)–7–Hexadecenal	56797–40–1	–	–	5.15 ± 0.08	–	–	–
36	Nonanal	124–19–6	54.61 ± 16.02 ^bc^	51.65 ± 9.69 ^bc^	48.38 ± 5.13 ^c^	65.01 ± 4.85 ^b^	120.54 ± 48.75 ^a^	95.47 ± 5.93 ^a^
37	(2E)–2–Octenal	2548–87–0	–	5.86 ± 0.46 ^d^	12.63 ± 1.88 ^bc^	9.69 ± 1.08 ^c^	21.24 ± 1.07 ^a^	13.03 ± 0.22 ^b^
38	Furfural	98–01–1	103.68 ± 44.66 ^a^	49.76 ± 4.53 ^b^	49.07 ± 0.99 ^b^	62.96 ± 13.81 ^b^	86.5 ± 9.31 ^a^	20.58 ± 2.01 ^c^
39	Benzaldehyde	100–52–7	125.37 ± 59.63 ^bc^	97.89 ± 10.11 ^c^	89.6 ± 8.26 ^c^	131.86 ± 23.24 ^b^	200.79 ± 10.8 ^a^	110.81 ± 37.91 ^bc^
40	Octanal	124–13–0	–	–	–	0.83 ± 0.09 ^a^	1.54 ± 0.71 ^a^	–
	**Lipids**							
41	Sec–butyl nitrite	924–43–6	–	–	–	–	–	20.1 ± 2.75
42	Vinyl acetate	108–05–4	34.38 ± 12.34 ^c^	37.02 ± 18.58 ^bc^	36.7 ± 5.57 ^c^	61.72 ± 10.87 ^ab^	60.01 ± 22.73 ^abc^	80.45 ± 20.18 ^a^
43	Ethyl butanoate	105–54–4	–	–	–	–	–	8.85 ± 2.95
44	Arachic acid benzyl ester	77509–04–7	4.12 ± 0.37 ^c^	4.56 ± 0.25 ^c^	5.06 ± 0.07 ^b^	5.96 ± 0.08 ^a^	–	–
45	Dodecan–2–yl 2,2,2–trifluoroacetate	1894–68–4	–	–	0.37 ± 0.13 ^a^	0.24 ± 0.01 ^a^	–	–
46	12,15–Octadecadiynoic acid methyl ester	57156–95–3	4.3 ± 1.34 ^b^	–	9.46 ± 0.33 ^a^	10 ± 1.24 ^a^	10.28 ± 3.3 ^a^	–
47	Ethyl hexanoate	123–66–0	1.83 ± 0.31 ^d^	4.1 ± 1.72 ^c^	5.99 ± 1.05 ^c^	11.26 ± 2.4 ^b^	20.53 ± 6.41 ^a^	21.79 ± 0.76 ^a^
48	Benzylcarbinyl caproate	6290–37–5	4.87 ± 1.1 ^d^	7.83 ± 0.24 ^c^	7.67 ± 1.19 ^c^	12.1 ± 0.65 ^b^	29.76 ± 4.43 ^a^	–
49	Dodecyl isobutyl carbonate	959067–22–2	–	–	–	–	15.57 ± 7.78	–
50	Ethyl caprylate	106–32–1	23.67 ± 6.96 ^e^	37.73 ± 0.21 ^d^	71.91 ± 7.48 ^c^	108.96 ± 4.74 ^b^	168.56 ± 3.7 ^a^	169.74 ± 22.12 ^a^
51	Hexyl hexanoate	6378–65–0	0.96 ± 0.34	–	–	–	–	–
52	Ethyl caprate	110–38–3	4.44 ± 0.05 ^c^	–	10.28 ± 1.3 ^b^	18.36 ± 3.42 ^a^	21.37 ± 5.7 ^a^	20.01 ± 3.09 ^a^
53	Acetic acid, 2–chloro–, nonyl ester	5451–96–7	–	–	–	5.67 ± 1.87 ^c^	10.94 ± 1.41 ^b^	16.31 ± 1.96 ^a^
54	Allyl 2–ethylbutyrate	7493–69–8	7.49 ± 2.73 ^bc^	3.98 ± 0.65 ^d^	6.21 ± 0.45 ^c^	13.12 ± 4.5 ^ab^	5.12 ± 1.42 ^cd^	14.16 ± 0.32 ^a^
55	Ethyl laurate	106–33–2	–	–	–	7.01 ± 3.81 ^a^	7.36 ± 3.71 ^a^	4.59 ± 2.2 ^b^
	**Ketones**							
56	2–Heptanone	110–43–0	–	6.15 ± 0.68 ^b^	–	–	–	51.32 ± 13.22 ^a^
57	2,3–Butanedione	431–03–8	–	–	70.84 ± 5.61 ^a^	–	58.75 ± 5.66 ^b^	–
58	Hydroxy Propanone	116–09–6	–	–	31.82 ± 4.6 ^b^	30.6 ± 6 ^b^	43.27 ± 6.12 ^a^	18.5 ± 0.54 ^c^
59	2–Nonanone	821–55–6	19.29 ± 3.85 ^cd^	19.69 ± 1.53 ^d^	22.13 ± 1.41 ^cd^	25.05 ± 2.61 ^c^	39.64 ± 3.36 ^b^	106 ± 16.41 ^a^
60	6–methyloct–5–en–2–one	24199–46–0	–	–	–	3.71 ± 0.23	–	–
61	2–Undecanone	112–12–9	11.73 ± 1.87 ^b^	0.79 ± 0.14 ^c^	11.38 ± 1.28 ^b^	12.93 ± 1.41 ^b^	16.98 ± 1.77 ^a^	14.4 ± 1.76 ^ab^
62	3,6–dimethyloctan–2–one	118452–32–7	–	–	2.59 ± 0.05	–	–	–
63	1–(3,5–di–tert–butyl–4–hydroxy–phenyl)–propan–1–one	14035–34–8	16.51 ± 4.22 ^ab^	12.53 ± 1.34 ^b^	12.56 ± 3.58 ^b^	19.16 ± 2.08 ^a^	19.93 ± 2.12 ^a^	–
	**Acids**							
64	3–hexenoic acid	4219–24–3	–	0.87 ± 0.02 ^a^	–	1.22 ± 0.46 ^a^	–	–
65	Acetic acid	64–19–7	462.9 ± 6.01 ^a^	288.04 ± 30.77 ^cd^	328.94 ± 20.03 ^c^	447.13 ± 22.18 ^ab^	419.33 ± 25.04 ^b^	225.18 ± 43.12 ^d^
66	Isobutyric acid	79–31–2	1.83 ± 0.38 ^c^	2.63 ± 1.24 ^c^	11.25 ± 3.06 ^ab^	5.61 ± 1.52 ^b^	13.07 ± 2.95 ^a^	–
67	4–hydroxybutyric acid	591–81–1	5.91 ± 0.02 ^d^	6.48 ± 1.21 ^c^	9.34 ± 1.33 ^b^	10.38 ± 1.76 ^b^	13.11 ± 0.41 ^a^	9.91 ± 2.04 ^b^
	**Others**							
68	2,3–dihydro–Furan	1191–99–7	–	–	–	–	106.64 ± 13.53	–
69	Limonene	138–86–3	15.23 ± 5.08 ^d^	47.56 ± 1.73 ^c^	54.16 ± 5.9 ^c^	73.44 ± 11.91 ^b^	85.69 ± 4.05 ^b^	107.2 ± 7.32 ^a^
70	2–Amylfuran	3777–69–3	3.89 ± 1.76 ^e^	7.59 ± 0.16 ^d^	8.65 ± 1.47 ^d^	11.05 ± 0.63 ^c^	41.59 ± 12.68 ^a^	16.11 ± 4.06 ^b^
71	Acetoin	513–86–0	710.51 ± 51.37 ^c^	605.65 ± 34.92 ^d^	589.9 ± 75.98 ^cd^	954.68 ± 144.19 ^b^	1377.89 ± 200.15 ^a^	1163.8 ± 211.1 ^ab^
72	2–Acetylthiazole	24295–03–2	3.37 ± 0.06 ^c^	1.85 ± 0.7 ^d^	3.33 ± 1.21 ^c^	1.25 ± 0.15 ^d^	7.55 ± 1.28 ^a^	5.48 ± 0.76 ^b^

Note: “–” means not detected. Results are expressed as mean ± standard deviation (n = 3); different letters are marked in the same row to indicate different significance levels (*p* < 0.05).

## Data Availability

Data is contained within the article or Supplementary Material.

## Supplementary Materials

The following supporting information can be downloaded at: https://www.mdpi.com/article/10.3390/foods13030461/s1, Table S1: Types of response substances corresponding to different sensors of PEN3 electronic nose; Table S2: Sensory scoring criteria; Table S3: Figure 4A,B Electronic Nose Sensor Response Values; Figure S1: GC–MS chromatogram of bread crust; Figure S2: GC–MS chromatogram of bread crumb.

## References

[1] Lynch K.M., Steffen E.J., Arendt E.K. (2016). Brewers’ spent grain: A review with an emphasis on food and health. J. Inst. Brew..

[2] Patrignani M., Brantsen J.F., Awika J.M., Conforti P.A. (2021). Application of a novel microwave energy treatment on brewers’ spent grain (BSG): Effect on its functionality and chemical characteristics. Food Chem..

[3] Baiano A., la Gatta B., Rutigliano M., Fiore A. (2023). Functional Bread Produced in a Circular Economy Perspective: The Use of Brewers’ Spent Grain. Foods.

[4] Czubaszek A., Wojciechowicz-Budzisz A., Spychaj R., Kawa-Rygielska J. (2022). Effect of Added Brewer’s Spent Grain on the Baking Value of Flour and the Quality of Wheat Bread. Molecules.

[5] Ye S., Shah B.R., Li J., Liang H., Zhan F., Geng F., Li B. (2022). A critical review on interplay between dietary fibers and gut microbiota. Trends Food Sci. Technol..

[6] Malavi D., Mbogo D., Moyo M., Mwaura L., Low J., Muzhingi T. (2022). Effect of Orange-Fleshed Sweet Potato Puree and Wheat Flour Blends on beta-Carotene, Selected Physicochemical and Microbiological Properties of Bread. Foods.

[7] Akin P.A., Sezer B., Sanal T., Apaydin H., Koksel H., Boyaci H. (2020). Multi-elemental analysis of flour types and breads by using laser induced breakdown spectroscopy. J. Cereal Sci..

[8] Cabello-Olmo M., Krishnan P.G., Araña M., Oneca M., Díaz J.V., Barajas M., Rovai M. (2023). Development, Analysis, and Sensory Evaluation of Improved Bread Fortified with a Plant-Based Fermented Food Product. Foods.

[9] Oyedeji A.B., Wu J. (2023). Food-based uses of brewers spent grains: Current applications and future possibilities. Food Biosci..

[10] Ganesan A.R., Hoellrigl P., Mayr H., Loesch D.M., Tocci N., Venir E., Conterno L. (2023). The Rheology and Textural Properties of Bakery Products Upcycling Brewers’ Spent Grain. Foods.

[11] Luo S., Hou Y., Xie L., Zhang H., Liu C., Chen T. (2023). Effects of microwave on the potential microbiota modulating effects of agro-industrial by-product fibers among different individuals. LWT.

[12] Wang L., Wang M., Zhou Y., Wu Y., Ouyang J. (2022). Influence of ultrasound and microwave treatments on the structural and thermal properties of normal maize starch and potato starch: A comparative study. Food Chem..

[13] Thiex N. (2009). Evaluation of Analytical Methods for the Determination of Moisture, Crude Protein, Crude Fat, and Crude Fiber in Distillers Dried Grains with Solubles. J. AOAC Int..

[14] Thiex N., Novotny L., Crawford A. (2012). Determination of Ash in Animal Feed: AOAC Official Method 942.05 Revisited. J. AOAC Int..

[15] Stojanovska L., Ali H.I., Kamal-Eldin A., Souka U., Al Dhaheri A.S., Ismail L.C., Hilary S. (2023). Soluble and Insoluble Dietary Fibre in Date Fruit Varieties: An Evaluation of Methods and Their Implications for Human Health. Foods.

[16] Chisenga S.M., Workneh T.S., Bultosa G., Alimi B.A., Siwela M. (2020). Dough rheology and loaf quality of wheat-cassava bread using different cassava varieties and wheat substitution levels. Food Biosci..

[17] Aljobair M.O. (2022). Effect of Chia Seed as Egg Replacer on Quality, Nutritional Value, and Sensory Acceptability of Sponge Cake. J. Food Qual..

[18] Tong S., Erqing Z., Yingting Y., Tao Z., Ting X., Lihong D., Fei H., Dongxiao S. (2023). Utilization of ovalbumin-ferulic acid-carrageenan Pickering emulsion in baked bread for butter reduction: Bread microstructural properties and quality. LWT.

[19] Shen H., Wei T., Zhang Z., Zheng Q., Guo R., Jiang H., Zhang G., Zheng J. (2020). Discrimination of five brands of instant vermicelli seasonings by HS-SPME/GC–MS and electronic nose. J. Food Sci. Technol..

[20] Zhang J., Pan L., Tu K. (2023). Aroma in freshly squeezed strawberry juice during cold storage detected by E-nose, HS–SPME–GC–MS and GC-IMS. J. Food Meas. Charact..

[21] Li C., Wan H., Wu X., Yin J., Zhu L., Chen H., Song X., Han L., Yang W., Yu H. (2022). Discrimination and Characterization of the Volatile Organic Compounds in *Schizonepetae Spica* from Six Regions of China Using HS-GC-IMS and HS-SPME-GC-MS. Molecules.

[22] Spaggiari M., Marchini M., Calani L., Dodi R., Di Pede G., Dall’asta M., Scazzina F., Barbieri A., Righetti L., Folloni S. (2022). Evolutionary Wheat Populations in High-Quality Breadmaking as a Tool to Preserve Agri-Food Biodiversity. Foods.

[23] Tóth M., Kaszab T., Meretei A. (2022). Texture profile analysis and sensory evaluation of commercially available gluten-free bread samples. Eur. Food Res. Technol..

[24] Behere M., Patil S.S., Rathod V.K. (2021). Rapid extraction of watermelon seed proteins using microwave and its functional properties. Prep. Biochem. Biotechnol..

[25] Meng S., Li J., Chang S., Maleki S.J. (2019). Quantitative and kinetic analyses of peanut allergens as affected by food processing. Food Chem. X.

[26] López-Linares J.C., García-Cubero M., Lucas S., González-Benito G., Coca M. (2019). Microwave assisted hydrothermal as greener pretreatment of brewer’s spent grains for biobutanol production. Chem. Eng. J..

[27] Sezer D.B., Ahmed J., Sumnu G., Sahin S. (2021). Green processing of sour cherry (*Prunus cerasus* L.) pomace: Process optimization for the modification of dietary fibers and property measurements. J. Food Meas. Charact..

[28] Stantiall S.E., Serventi L. (2018). Nutritional and sensory challenges of gluten-free bakery products: A review. Int. J. Food Sci. Nutr..

[29] Talens C., Arboleya J.C., Castro-Giraldez M., Fito P.J. (2017). Effect of microwave power coupled with hot air drying on process efficiency and physico-chemical properties of a new dietary fibre ingredient obtained from orange peel. LWT.

[30] de Erive M.O., He F., Wang T., Chen G. (2020). Development of beta-glucan enriched wheat bread using soluble oat fiber. J. Cereal Sci..

[31] Wang C.-C., Yang Z., Guo X.-N., Zhu K.-X. (2021). Effects of insoluble dietary fiber and ferulic acid on the quality of steamed bread and gluten aggregation properties. Food Chem..

[32] Aleixandre A., Benavent-Gil Y., Velickova E., Rosell C.M. (2021). Mastication of crisp bread: Role of bread texture and structure on texture perception. Food Res. Int..

[33] Wirkijowska A., Zarzycki P., Sobota A., Nawrocka A., Blicharz-Kania A., Andrejko D. (2019). The possibility of using by-products from the flaxseed industry for functional bread production. LWT.

[34] Han A., Romero H.M., Nishijima N., Ichimura T., Handa A., Xu C., Zhang Y. (2019). Effect of egg white solids on the rheological properties and bread making performance of gluten-free batter. Food Hydrocoll..

[35] Wiedemair V., Gruber K., Knöpfle N., Bach K.E. (2022). Technological Changes in Wheat-Based Breads Enriched with Hemp Seed Press Cakes and Hemp Seed Grit. Molecules.

[36] Franco M., Belorio M., Gómez M. (2022). Assessing Acerola Powder as Substitute for Ascorbic Acid as a Bread Improver. Foods.

[37] Cantero L., Salmerón J., Miranda J., Larretxi I., Fernández-Gil M.d.P., Bustamante M., Matias S., Navarro V., Simón E., Martínez O. (2022). Performance of Apple Pomace for Gluten-Free Bread Manufacture: Effect on Physicochemical Characteristics and Nutritional Value. Appl. Sci..

[38] Su J., Geng Y., Yao J., Huang Y., Ji J., Chen F., Hu X., Ma L. (2022). Quinone-mediated non-enzymatic browning in model systems during long-term storage. Food Chem. X.

[39] Amoriello T., Mellara F., Galli V., Amoriello M., Ciccoritti R. (2020). Technological Properties and Consumer Acceptability of Bakery Products Enriched with Brewers’ Spent Grains. Foods.

[40] Debonne E., Van Bockstaele F., Philips E., De Leyn I., Eeckhout M. (2017). Impact of par-baking and storage conditions on the quality of par-baked and fully baked bread. LWT.

[41] Ktenioudaki A., Chaurin V., Reis S.F., Gallagher E. (2012). Brewer’s spent grain as a functional ingredient for breadsticks. Int. J. Food Sci. Technol..

[42] Ting X., Shuying T., Tong S., Zhenzhen H., Fei H., Ruifen Z., Lihong D., Mei D., Yingbin S., Dongxiao S. (2022). Impact of replacing wheat flour with lychee juice by-products on bread quality characteristics and microstructure. LWT.

[43] Ozkoc S.O., Sumnu G., Sahin S. (2009). The effects of gums on macro and micro-structure of breads baked in different ovens. Food Hydrocoll..

[44] Leggieri M.C., Mazzoni M., Fodil S., Moschini M., Bertuzzi T., Prandini A., Battilani P. (2021). An electronic nose supported by an artificial neural network for the rapid detection of aflatoxin B_1_ and fumonisins in maize. Food Control.

[45] Fărcaş A.C., Socaci S.A., Dulf F.V., Tofană M., Mudura E., Diaconeasa Z. (2015). Volatile profile, fatty acids composition and total phenolics content of brewers’ spent grain by-product with potential use in the development of new functional foods. J. Cereal Sci..

[46] Wang S., Xu X., Wang S., Wang J., Peng W. (2022). Effects of Microwave Treatment on Structure, Functional Properties and Antioxidant Activities of Germinated Tartary Buckwheat Protein. Foods.

[47] Jian-Li Y., Qing-An Z., Meng-Jia L. (2021). Utilization of apricot kernel skins by ultrasonic treatment of the dough to produce a bread with better flavor and good shelf life. LWT.

[48] Zhang K., Zhang C., Gao L., Liu Y. (2022). Microbial diversity in laomian and yeast dough and its influence on volatiles in Chinese-steamed bread. J. Food Process. Preserv..

[49] Chang X., Huang X., Tian X., Wang C., Aheto J.H., Ernest B., Yi R. (2020). Dynamic characteristics of dough during the fermentation process of Chinese steamed bread. Food Chem..

[50] Labanska M., van Amsterdam S., Jenkins S., Clarkson J.P., Covington J.A. (2022). Preliminary Studies on Detection of Fusarium Basal Rot Infection in Onions and Shallots Using Electronic Nose. Sensors.

[51] Khoozani A.A., Kebede B., Bekhit A.E.-D.A. (2022). The effects of green banana flour fortification on volatiles compounds of bread: A fingerprinting approach. Appl. Food Res..

[52] Pico J., Martínez M.M., Bernal J., Gómez M. (2017). Impact of frozen storage time on the volatile profile of wheat bread crumb. Food Chem..

[53] Yaling H., Junwen W., Min S., Tao F., Qian L., Shiqing S., Xiaoming Z., Chi-Tang H. (2023). Flavor profile disclosure of Chinese steamed breads (CSBs) by sensomics approach. Food Biosci..

[54] Ktenioudaki A., Crofton E., Scannell A.G.M., Hannon J.A., Kilcawley K.N., Gallagher E. (2013). Sensory properties and aromatic composition of baked snacks containing brewer’s spent grain. J. Cereal Sci..

[55] Wu S., Peng Y., Xi J., Zhao Q., Xu D., Jin Z., Xu X. (2022). Effect of sourdough fermented with corn oil and lactic acid bacteria on bread flavor. LWT.

[56] Izzreen M.N.N.Q., Petersen M.A., Hansen S. (2016). Volatile Compounds in Crumb of Whole-Meal Wheat Bread Fermented with Different Yeast Levels and Fermentation Temperatures. Cereal Chem..

[57] Farcas A.C., Socaci S.A., Chiș M.S., Pop O.L., Fogarasi M., Păucean A., Igual M., Michiu D. (2021). Reintegration of Brewers Spent Grains in the Food Chain: Nutritional, Functional and Sensorial Aspects. Plants.

[58] Jensen S., Oestdal H., Skibsted L.H., Larsen E., Thybo A.K. (2011). Chemical changes in wheat pan bread during storage and how it affects the sensory perception of aroma, flavour, and taste. J. Cereal Sci..

[59] Birch A.N., Petersen M.A., Hansen Å.S. (2013). The aroma profile of wheat bread crumb influenced by yeast concentration and fermentation temperature. LWT Food Sci. Technol..

[60] Barbarisi C., De Vito V., Pellicano M.P., Boscaino F., Balsamo S., Laurino C., Sorrentino G., Volpe M.G. (2019). Bread chemical and nutritional characteristics as influenced by food grade sea water. Int. J. Food Prop..

[61] Shih Y.-T., Wang W., Hasenbeck A., Stone D., Zhao Y. (2020). Investigation of physicochemical, nutritional, and sensory qualities of muffins incorporated with dried brewer’s spent grain flours as a source of dietary fiber and protein. J. Food Sci..

