# Supplementary material for: Study on the Effect of Microwaved Brewer’s Spent Grains on the Quality and Flavor Characteristics of Bread
Source: Foods. 2024 Feb 1;13(3):461. doi: 10.3390/foods13030461 (PMC10855328; doi:10.3390/foods13030461)
Supplement: Supplementary file 1 [file foods-13-00461-s001.zip › foods-2723559-supplementary.pdf]

Supplementary Material

Table S1. Types of response substances corresponding to different sensors of PEN3 electronic nose.

Number	Sensor type	Performance description
1	W1C	Aromatic components, benzene
2	W5S	Sensitive and sensitive to nitrogen oxides
3	W3C	Ammonia, sensitive to aromatic components
4	W6S	Hydride (hydrogen)
5	W5C	Short-chain alkane aromatic components
6	W1S	Sensitive to methyl groups (methane)
7	W1W	Sensitive to inorganic sulfides
8	W2S	Sensitive to alcohols, aldehydes and ketones (ethanol)
9	W2W	Aromatic components, sensitive to organic sulfides
10	W3S	Sensitive to alkanes (long chain alkanes)

Table S2. Sensory scoring criteria.

Grade items	Scoring rules	Score
Crust color	Homogeneous, shiny, yellowish brown	7-9
	More uniform, slightly shiny, light yellow or tan	4-6
	Uneven, dull, whitish or dark brown	1-3
Crust texture	No collapse folds, no cracks, soft and tender	7-9
	Collapse folds are few, no cracks, moderate thickness and hardness	4-6
	Collapsed folds are more numerous, cracked, thicker and harder	1-3
Crumb color	Homogeneous, shiny, creamy yellow	7-9
	More uniform, shiny, creamy white	4-6
	Uneven, dull, off-white	1-3
Crumb structure	The shape is full and complete, the pressure is elastic, the stomata are uniform and evenly distributed	7-9
	The shape is full and complete, the pressure is elastic, there are individual large pores, the distribution of stomata is more uniform	4-6
	The shape is damaged, the pressure is inelastic, the pores are large and unevenly distributed	1-3
Mouthfeel	Soft, chewy, sticky but not sticky	7-9
	Softer, chewier, sticky but not sticky	4-6
	Dry hard, non-sticky, dross	1-3
Taste	Moderate milk taste and sweetness, no acid, bitter and other peculiar smells	7-9
	There is almost no milky and sweet taste, no acid, bitterness and other peculiar smells	4-6
	There is almost no milky and sweet taste, and there are peculiar smells such as sour and bitter	1-3
Aroma	It has a strong malt aroma of roasting and fermentation, and no sour odor	7-9
	Roasted and fermented malt has a light aroma and no peculiar smells such as acid odor	4-6
	There is almost no roasting and fermentation malt aroma, sour odor and other peculiar smells	1-3
Overall preference	9 points are extreme like, 8 is very like, 7 is general like, 6 is a little like, 5 is both like and dislike, 4 is a little dislike, 3 is general dislike, 2 is very disliked, 1 is extreme dislike	

Table S3. Figure 4 A and B Electronic Nose Sensor Response Values.

MW-BSG(%)		Sensors									
		W1C	W5S	W3C	W6S	W5C	W1S	W1W	W2S	W2W	W3S
Crust	0	3.13 ± 0.31^{e}	10.19 ± 1.16^{e}	2.12 ± 0.13^{f}	1.14 ± 0.03^{c}	1.55 ± 0.07^{f}	8.06 ± 1^{d}	4.5 ± 0.28^{d}	6.83 ± 0.91^{e}	1.24 ± 0.02^{d}	1.21 ± 0.03^{d}
	2	3.68 ± 0.19^{d}	12.43 ± 0.03^{d}	2.44 ± 0.01^{e}	1.32 ± 0.02^{ab}	1.73 ± 0.01^{e}	12.43 ± 0.53^{c}	4.21 ± 0.15^{d}	12.23 ± 0.42^{d}	1.22 ± 0.01^{d}	1.51 ± 0.01^{b}
	4	4.15 ± 0.22^{c}	14.37 ± 0.28^{c}	2.7 ± 0.07^{d}	1.24 ± 0.1^{b}	1.91 ± 0.06^{d}	12.06 ± 0.29^{c}	5.44 ± 0.32^{c}	12.34 ± 0.36^{cd}	1.32 ± 0.01^{c}	1.4 ± 0.03^{c}
	6	5.98 ± 0.28^{a}	21.87 ± 0.97^{a}	3.65 ± 0.13^{a}	1.38 ± 0.03^{a}	2.54 ± 0.09^{a}	19.56 ± 1.37^{a}	7.89 ± 0.27^{a}	21.04 ± 1.61^{a}	1.51 ± 0.02^{a}	1.59 ± 0.03^{a}
	8	4.93 ± 0.2^{b}	20.58 ± 0.63^{a}	3.14 ± 0.08^{b}	1.34 ± 0.03^{ab}	2.23 ± 0.04^{b}	15.88 ± 0.58^{b}	7.94 ± 0.27^{a}	16.68 ± 0.59^{b}	1.48 ± 0.04^{a}	1.54 ± 0.06^{ab}
	10	4.47 ± 0.18^{c}	16.43 ± 0.33^{b}	2.93 ± 0.08^{c}	1.28 ± 0.01^{b}	2.1 ± 0.06^{c}	13.15 ± 0.35^{c}	6.66 ± 0.13^{b}	14.05 ± 0.61^{c}	1.38 ± 0.01^{b}	1.47 ± 0.06^{bc}
Crumb	0	6.28 ± 0.3^{ab}	21.1 ± 1.26^{b}	3.7 ± 0.17^{a}	1.41 ± 0.01^{a}	2.53 ± 0.12^{ab}	23.53 ± 1.67^{a}	6.99 ± 0.49^{b}	24.34 ± 0.98^{b}	1.43 ± 0.03^{c}	1.64 ± 0.02^{a}
	2	4.87 ± 0.22^{d}	16.28 ± 0.31^{d}	3 ± 0.05^{c}	1.38 ± 0.01^{b}	2.07 ± 0.03^{d}	16.38 ± 0.16^{c}	5.16 ± 0.25^{c}	17.87 ± 0.27^{d}	1.29 ± 0.01^{d}	1.57 ± 0.04^{b}
	4	6.6 ± 0.19^{a}	23.12 ± 0.71^{a}	3.9 ± 0.16^{a}	1.36 ± 0.01^{b}	2.65 ± 0.13^{a}	22.9 ± 1.01^{a}	8.29 ± 0.21^{a}	26.46 ± 0.96^{a}	1.51 ± 0.01^{ab}	1.67 ± 0.03^{a}
	6	6.11 ± 0.23^{b}	22.37 ± 0.15^{a}	3.79 ± 0.06^{a}	1.38 ± 0.01^{b}	2.68 ± 0.02^{a}	18.99 ± 0.34^{b}	8.64 ± 0.38^{a}	21.9 ± 0.71^{c}	1.53 ± 0.03^{a}	1.56 ± 0.03^{b}
	8	5.3 ± 0.14^{c}	20.17 ± 0.27^{b}	3.38 ± 0.07^{b}	1.29 ± 0.01^{c}	2.41 ± 0.07^{bc}	15.71 ± 0.34^{cd}	8.65 ± 0.48^{a}	17.39 ± 0.4^{d}	1.48 ± 0.02^{b}	1.44 ± 0.03^{c}
	10	5.06 ± 0.03^{cd}	17.57 ± 0.3^{c}	3.28 ± 0.02^{b}	1.25 ± 0.02^{d}	2.36 ± 0.02^{c}	14.56 ± 0.41^{d}	7.2 ± 0.19^{b}	15.79 ± 0.09^{e}	1.41 ± 0.01^{c}	1.48 ± 0.02^{c}

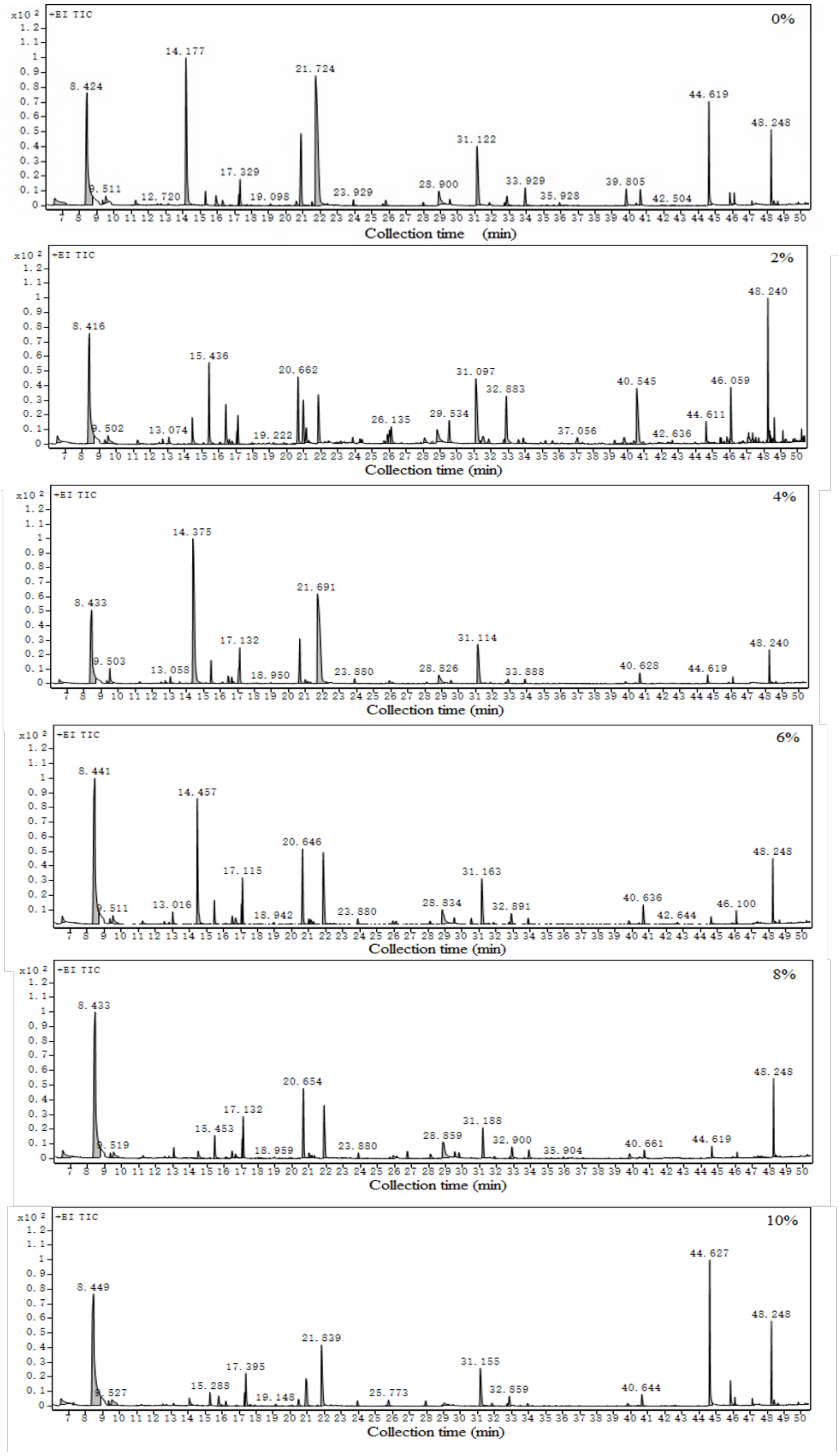

Figure S1. GC-MS chromatogram of bread crust.

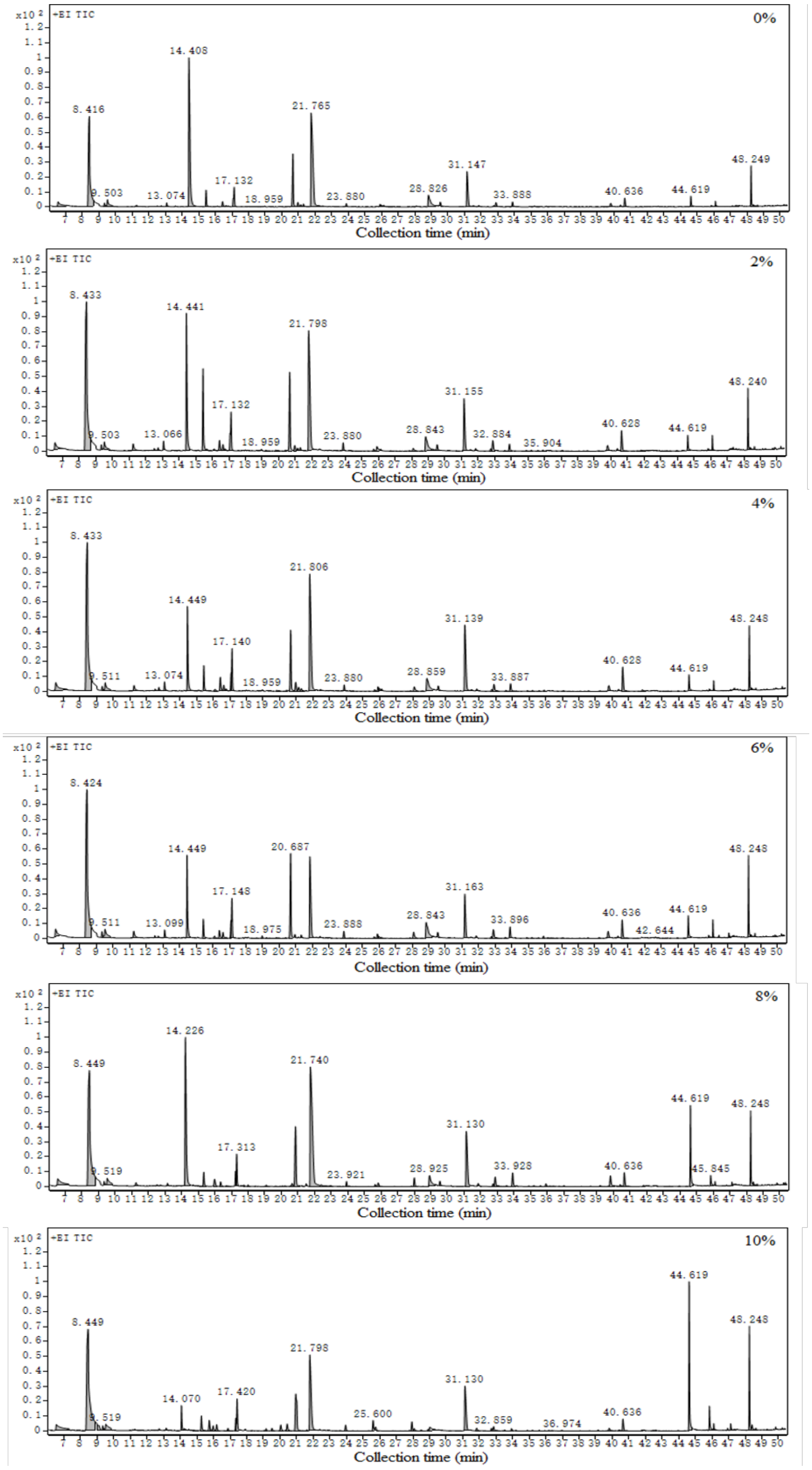

Figure S2. GC-MS chromatogram of bread crumb.
